# Exosomes‐Loaded Electroconductive Hydrogel Synergistically Promotes Tissue Repair after Spinal Cord Injury via Immunoregulation and Enhancement of Myelinated Axon Growth

**DOI:** 10.1002/advs.202105586

**Published:** 2022-03-06

**Authors:** Lei Fan, Can Liu, Xiuxing Chen, Lei Zheng, Yan Zou, Huiquan Wen, Pengfei Guan, Fang Lu, Yian Luo, Guoxin Tan, Peng Yu, Dafu Chen, Chunlin Deng, Yongjian Sun, Lei Zhou, Chengyun Ning

**Affiliations:** ^1^ School of Materials Science and Engineering and National Engineering Research Center for Tissue Restoration and Reconstruction South China University of Technology No. 381, Wushan Road, Tianhe District Guangzhou 510641 China; ^2^ Department of Orthopedic Surgery The First Affiliated Hospital Zhejiang University School of Medicine Hangzhou 310003 China; ^3^ Guangdong Provincial Key Laboratory of Malignant Tumor Epigenetics and Gene Regulation Department of Medical Oncology Sun Yat‐sen Memorial Hospital Sun Yat‐sen University No. 107, Yanjiang West Road, Yuexiu District, Guangzhou Guangzhou 510120 China; ^4^ Laboratory Medicine Center Nanfang Hospital Southern Medical University No. 1838, Guangzhou Avenue North, Baiyun District Guangzhou Guangdong 510515 China; ^5^ Department of Radiology the Third Affiliated Hospital of Sun Yat‐sen University No. 600, Tianhe Road, Tianhe District Guangzhou 510630 China; ^6^ Department of Pediatric Orthopedic Center for Orthopedic Surgery The Third Affiliated Hospital of Southern Medical University No.183, Zhongshan Avenue West Guangzhou 510515 China; ^7^ School of Preclinical Medicine Beijing University of Chinese Medicine No.11, North Third Ring East Road, Chaoyang District Beijing 100029 China; ^8^ School of Chemical Engineering and Light Industry Guangdong University of Technology No.100, Waihuan West Road, Panyu District Guangzhou 510006 China; ^9^ Laboratory of Bone Tissue Engineering Beijing Research Institute of Orthopaedics and Traumatology Beijing JiShuiTan Hospital No.31, Xinjiekou East Street, Xicheng District Beijing 100035 China; ^10^ Guangzhou Key Laboratory of Spine Disease Prevention and Treatment Department of Spine Surgery The Third Affiliated Hospital Guangzhou Medical University No. 63, Duobao Road, Liwan District Guangzhou 510150 China

**Keywords:** anti‐inflammation, axonal regeneration, BMSC‐derived exosomes, electroconductive hydrogels, spinal cord injury

## Abstract

Electroconductive hydrogels are very attractive candidates for accelerated spinal cord injury (SCI) repair because they match the electrical and mechanical properties of neural tissue. However, electroconductive hydrogel implantation can potentially aggravate inflammation, and hinder its repair efficacy. Bone marrow stem cell‐derived exosomes (BMSC‐exosomes) have shown immunomodulatory and tissue regeneration effects, therefore, neural tissue‐like electroconductive hydrogels loaded with BMSC‐exosomes are developed for the synergistic treatment of SCI. These exosomes‐loaded electroconductive hydrogels modulate microglial M2 polarization via the NF‐*κ*B pathway, and synergistically enhance neuronal and oligodendrocyte differentiation of neural stem cells (NSCs) while inhibiting astrocyte differentiation, and also increase axon outgrowth via the PTEN/PI3K/AKT/mTOR pathway. Furthermore, exosomes combined electroconductive hydrogels significantly decrease the number of CD68‐positive microglia, enhance local NSCs recruitment, and promote neuronal and axonal regeneration, resulting in significant functional recovery at the early stage in an SCI mouse model. Hence, the findings of this study demonstrate that the combination of electroconductive hydrogels and BMSC‐exosomes is a promising therapeutic strategy for SCI repair.

## Graphical abstract

Neural tissue‐like electroconductive hydrogels loaded with bone marrow stem cell‐derived exosomes are developed. The mechanism of the exosomes‐loaded electroconductive hydrogel synergistically regulating neural stem cell differentiation is explored. The exosomes‐combined electroconductive hydrogels significantly decrease inflammation reaction, enhance local neurogenesis, and promote axonal regeneration, resulting in significant functional recovery at the early stage in a spinal cord injury mouse model.

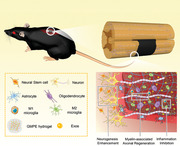

## 1 Introduction

An estimated 27 million people live with long‐term disability following spinal cord injury (SCI) worldwide, with ≈180 000 new cases occurring each year.^[^ ^1^ ^]^ SCI is followed by neuronal loss and axon and myelin necrosis, which leads to an extensive inflammatory response and further exacerbates secondary damage.^[^ ^2^ ^]^ Meanwhile, activated inflammatory cells (microglia) release proinflammatory cytokines, which contribute to reactive astrocyte gathering, subsequently increasing the release of inhibitory molecules such as chondroitin sulfate proteoglycans (CSPGs) at the injury site.^[^ ^3^ ^]^ Because of the inflammatory microenvironment and limited neural regeneration capacity, the injury is resolved by the formation of a dense glial scar, which acts as a barrier to neural and axonal regeneration.^[^ ^4^ ^]^ Thus, modulation of the inflammatory microenvironment, enhancement of neural stem cell (NSCs) recruitment and neuronal regeneration, and guidance and promotion of myelinated axon growth are needed for treating spinal cord injury (SCI).

Decompressive surgery with re‐establishment of spinal stability and high‐dose intravenous methylprednisolone sodium succinate (MPSS) usage in the acute phase of injury (≤8 h) are the most common clinical treatments at present.^[^ ^5^ ^]^ However, the former only aims to avoid further secondary damage by relieving the pressure on the injured spinal cord, and the latter can reduce early inflammatory responses but with severe complications, while neither show the ability to promote axonal and neural regeneration and therefore, have limited therapeutic effectiveness. In this regard, experimental approaches to promote axon growth including cellular transplantation and scaffold biomaterials have been applied in SCI repair.^[^ ^6^ ^]^ Cellular transplantation has been used experimentally in clinical conditions with some success but continues to be limited by uncontrolled cell differentiation, low survival rates, ethical issues, and the inevitable cell loss after implantation.^[^ ^7^ ^]^ Considering the seriousness of these problems, cost‐effective and cell‐free biomaterial implants are highly desirable.

Scaffold biomaterial‐based therapy with tunable modulus, topology, patterned surfaces has been proposed as a strategy to promote neural tissue regeneration by providing 3D matrices with the desired biological, chemical, and physical characteristics that favor cellular attachment, growth, differentiation, and neurite extension.^[^ ^8^, ^9^ ^]^ In particular, since the soft and hydrated forms of hydrogels are similar to native nerve tissue, they are widely used to promote cellular growth and tissue formation after SCI.^[^ ^10^, ^11^ ^]^ Our previous study revealed that the mechanical properties of the hydrogel could modulate the fates of NSCs which are sensitive to mechanical changes. NSCs were inclined to differentiate into neurons and lengthen axons in soft scaffolds; otherwise, NSCs are prone to differentiate into astrocyte in slightly stiffer materials.^[^ ^12^, ^13^ ^]^ However, hydrogels with poor conductivity limit their application in regulating the function of excitable cell types such as muscle and nerve cells.^[^ ^11^ ^]^ Importantly, mimicking the electrical transmission properties of the native nerve tissue is highly beneficial for SCI repair. Currently, electroconductive hydrogels have emerged as a promising class of hydrogel scaffolds combining a hydrophilic matrix with conducting components such as electroconductive polymers, metallic nanoparticles, or carbon materials.^[^ ^12^ ^]^ Due to its tissue‐like softness and the inherent presence of electrical fields similar to the innate nervous system, the electroconductive hydrogel can provide mechanical and electrical cues for enhancing neuronal differentiation of NSCs and controlling neurite extension.^[^ ^11^, ^13^ ^]^ We previously developed a porous, highly electroconductive, soft, and biocompatible conducting polymer hydrogel, which forms a freestanding electroconductive hydrogel for implantation into the spinal cord hemisection gap, and recently demonstrated that implanting this electroconductive hydrogel after SCI stimulated endogenous NSCs recruitment and neuronal differentiation after SCI.^[^ ^14^, ^15^, ^16^ ^]^ Although electroconductive hydrogels effectively enhanced neuronal and axonal regeneration, their efficacy is compromised by host recognition and the subsequent foreign body immune responses, which cannot attenuate or even aggravate the early secondary inflammation after acute SCI.^[^ ^17^, ^18^ ^]^ Thus, single transplantation of electroconductive hydrogel may not be sufficient to achieve a substantial improvement in SCI repair.

Bone marrow stem cell (BMSC) therapy shows characteristic immunomodulatory effects and has been applied in severe clinical inflammatory diseases such as pancreatitis, colitis, and focal cerebral ischemia.^[^ ^15^ ^]^ Exosomes are involved in intercellular communication and act on the innate immune system as paracrine messengers. They also exert immunomodulatory effects and can alleviate immune abnormalities.^[^ ^19^, ^20^ ^]^ Recently, BMSC‐exosomes have emerged as a new cell‐free therapeutic platform for various diseases due to their therapeutic effects, including their ability to promote regeneration and modulate immunoreaction.^[^ ^21^ ^]^ BMSC‐exosomes, as a promising nanocarriers, carry various therapeutic growth factors and miRNAs, leading to axonal regeneration and angiogenesis enhancement, structural and electrophysiological improvements, and neuroinflammation and gliosis reduction, resulting in significant motor improvement and sensory recovery after spinal cord injury.^[^ ^21^, ^22^ ^]^ Meanwhile, neuroinflammation reduction caused by miRNAs contained in BMSC‐exosomes can negatively regulate the TLR4/NF‐*κ*B pathway.^[^ ^23^, ^24^ ^]^ Delivery of immune‐modulating BMSC‐exosomes in electroconductive hydrogel can attenuate adverse host immune response and exert synergistically therapeutic effects in combination with electroconductive hydrogel to promote functional recovery. However, exosome‐loaded electroconductive hydrogel systems have not been investigated for their ability to promote tissue repair. In view of this, we hypothesized that an electroconductive hydrogel combined with BMSC‐exosomes might achieve adequate therapeutic effectiveness in patients with SCI. Moreover, the delivery of BMSC‐exosomes in electroconductive hydrogels can attenuate adverse host immune effects while synergistically exerting the therapeutic effect of promoting neuronal and axonal regeneration, thereby alleviating SCI. Herein, we developed exosome‐loaded dual‐network electroconductive hydrogels composed of photo‐cross‐linkable gelatin methacrylate (GM) hydrogels and polypyrrole (PPy) hydrogels. First, the GM/PPy (GMP) hydrogel scaffold was fabricated by in situ growth of the PPy network cross‐linked and doped by tannic acid (TA) within the GM hydrogel networks, which possesses natural cell binding motifs such as Arg–Gly–Asp (RGD), allowing cells to grow within it. Then, BMSC‐exosomes were immobilized in the TA‐doped GMP hydrogel network to form a GM/PPy/exosomes (GMPE) hydrogel via reversible interactions formation due to the presence of large amounts of polyphenol groups in TA. The noncovalent binding did not affect the structure and bioactivity of the exosomes while ensuring a slow sustained release of exosomes early in the implantation. Cell biocompatibility, adhesion, growth, and differentiation on the GMPE hydrogel scaffold were evaluated in vitro. In addition, the specific signaling pathways via which GMPE hydrogels manipulate immune response and promote axonal regeneration were identified. A mouse spinal cord hemisection model was established to detect whether the GMPE hydrogel was efficient in facilitating nerve regeneration and improving functional recovery after SCI.

## 2 Results

### 2.1 Identification of BMSCs and BMSC‐Exosomes

Since nanoparticle tracking analysis (NTA) assay is a well‐established technique to quantify the concentration of exosomes according to MISEV2018 guidelines,^[^ ^25^ ^]^ we have conducted NTA assay to confirm that exosome‐free fatal bovine serum (FBS) successfully obtained by ultracentrifugation (Figure S1A, Supporting Information). The exosome‐free media was used as the cell cultures to isolate BMSC‐exosomes. The extraction of BMSCs and BMSC‐exosomes is illustrated in Figure S1B of the Supporting Information. Photomicrographs showed that primary BMSCs typically exhibited spindle‐like morphology (Figure S1C, Supporting Information). Flow cytometry showed that the obtained cells expressed high levels of the positive BMSCs surface markers such as CD29, CD90, and CD44H, but did not express negative surface markers such as CD11b and CD45 of BMSCs (Figure S1C, Supporting Information). These cells also successfully underwent adipogenic, osteogenic, and chondrogenic differentiation (Figure S1D, Supporting Information), which indicated the successful extraction of BMSCs. The presence of a cup‐shaped morphology was observed with transmission electron microscopy (TEM) analysis (Figure S1E, Supporting Information), a particle size of 70 to 140 nm from NTA devices (Figure S1F, Supporting Information), and the expression of Flotillin‐1, CD63, and TSG101 on the nanoparticles surface (Figure S1G, Supporting Information) all demonstrated successful extraction of BMSC‐derived exosomes. We have performed proteomic mass spectrometry to analyze the purity of the collected exosomes on the basis of the standards suggested by the International Society for Extracellular Vesicles (ISEV).^[^ ^19^ ^]^ The result showed that the absence of cellular or serum contaminants (Table S1, Supporting Information).

### 2.2 Fabrication of GM, GMP, and GMPE Hydrogels

**Figure 1:**
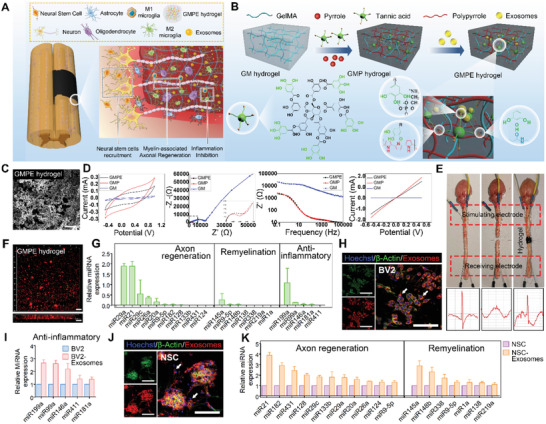
Characteristics of the GMPE hydrogels. A) Illustration of how the GMPE hydrogel can reduce early inflammation, enhance NSCs recruitment and promote myelin‐associated axonal regrowth to synergistically promote locomotor recovery after spinal cord hemisection. B) The three‐step synthesis procedure for the GMPE hydrogel was illustrated. The GMP hydrogel was synthesized by TA interacting with the amide bond on the GM backbone and the nitrogen groups on PPy chains. BMSC‐exosomes were reversibly immobilized into GMP hydrogels via hydrogen bond formation between TA polyphenol groups and phosphate groups in exosomes phospholipid to form GMPE hydrogel. C) Microstructure of the GMPE hydrogel was observed by SEM. Scale bars: 25 µm. D) Electrical characterization, including CV, EIS, *I*–*V*, and Bode plots of GMP and GMPE hydrogels showed excellent electrical performance. E) After the isolation of transected spinal cords, the stimulating electrical signals were retransmitted by GMPE hydrogels. F) IF imaging showed that exosomes were evenly distributed into the GMPE hydrogel and the penetration depth of the exosomes was more than 100 µm. Scale bars: 100 µm. G) RT‐qPCR indicated that BMSC‐exosomes express of axonal regeneration‐related, remyelination‐related, and anti‐inflammatory‐related miRNAs (*n* = 3). H) BV2 cells cultured on the GMPE hydrogel can normally phagocytize exosomes released from the hydrogel. White arrows indicate where BV2 cells have phagocytosed exosomes. Scale bars: 100 µm. I) Anti‐inflammatory‐related miRNAs expression increased as the result of BV2 cells phagocytosing exosomes (*n* = 3). J) PKH26‐labeled exosomes were clearly detected in the cytoplasm of NSCs, suggesting successful in vitro endocytosis of exosomes released from the GMPE hydrogel. White arrows indicate where NSCs have phagocytosed exosomes. Scale bars: 100 µm. K) Axonal regeneration‐related and remyelination‐related miRNAs expression increased after NSCs phagocytize exosomes (*n* = 3).

We used a three‐step synthesis process to produce GMPE hydrogels (**Figure** **1**A,B). First, the GM hydrogel networks were formed by ultraviolet (UV) light photo‐cross‐linking of GM units. Secondly, the GM hydrogel was successively immersed into solution I containing the monomers Py and TA and solution II containing ammonium persulfate (APS), allowing in situ polymerization and cross‐linking of conducting PPy chains. Transparent GM hydrogels become opaque with the formation of the PPy (black color), demonstrating successful polymerization of polypyrrole in the GM hydrogel (Figure S2A,B, Supporting Information). TA is an abundant natural water‐soluble polyphenol present in plants that can act as a dopant and cross‐linker for PPy hydrogel formation.^[^ ^11^ ^]^ In this study, TA interacted with the amide bond on the GM backbone via hydrogen bonds and also reacted with PPy chains by protonating the nitrogen groups on PPy to form a dual‐network electroconductive hydrogel (GMP hydrogel) with strong interactions. This GM network conferred biocompatibility, tissue‐like softness, degradability, and tissue and cell adhesion, while the PPy network provided electroconductive electrical activity to the hydrogels. Finally, the BMSC‐exosomes were immobilized into the GMP hydrogel network to form the GMPE hydrogel via reversible reversible interactions formation between the presence of a large amount of polyphenol groups in TA and the phosphate groups in the phospholipid of exosomes.

### 2.3 Characteristics of GM, GMP, and GMPE Hydrogel

In comparison with gelatin, GM showed two new peaks at 5.3 and 5.5 ppm that were attributed to the two protons of its methacrylate groups (Figure S2C, Supporting Information). The GM hydrogel exhibited amide bands characteristic of gelatin, including C═O stretching at 1650 cm^−1^ (amide I), C—N at 1440 cm^−1^, and N—H deformation at 1239 cm^−1^ (amide III). In the PPy spectrum, the peaks at 1556 and 1403 cm^−1^ are Py ring vibrations. These peaks also appeared in the spectra of the GMP hydrogels, indicating that the PPy chain was successfully incorporated into the GM hydrogel backbone (Figure S2D, Supporting Information). Scanning electron microscopy (SEM) analysis showed that GMPE hydrogels exhibited a 3D highly porous structure (Figure 1C, Supporting Information), which provided space for nerve cell extension. In addition, high‐magnification SEM also showed the interconnected globular nanoparticle morphology of PPy was coated onto the GM backbone (Figure S2E, Supporting Information). The mechanical properties of all samples were tested using dynamic oscillatory frequency sweep measurements. The storage moduli (elastic modulus, *G*′) of all hydrogels were larger than the loss moduli (viscous modulus, *G*″) over an angular frequency range of 1–100 Hz, indicating that the hydrogels had good stability (Figure S2F, Supporting Information). The average storage modulus at a 10 Hz angular frequency increased from 555.7 ± 50.1 Pa for the GM hydrogel to 1039.3 ± 89.3 Pa and 1056.0 ±133.1 Pa for the GMP and GMPE hydrogels, respectively (Figure S2G, Supporting Information). However, the mechanical properties of all three hydrogels matched neural tissue mechanics (600–3000 Pa), which was beneficial for cell function and differentiation. The introduction of hydrophobic PPy reduced the swelling ratio in the GMPE and GMP hydrogels when compared with GM hydrogel, although the difference was not statistically significant (Figure S2H, Supporting Information). Also, introducing TA may also contribute the mechanical strength enhancement and the swelling ratio reduction because of the strong interactions between polymers and the phenol groups in the TA molecules.^[^ ^26^ ^]^ Additionally, after soaking the hydrogel in the physiological medium for 7 and 14 days, the swelling ratio and mechanical properties of the GMPE hydrogel did not change significantly (Figure S2I–J, Supporting Information), indicating that the hydrogel exhibited long‐term swelling effect and mechanical stability. Ex vivo spinal cords were able to stick to the GMPE hydrogel, which indicated that the hydrogels also had excellent bioadhesion and supported their potential use in an in vivo animal SCI model (Figure S2K, Supporting Information). The plateau value of force/width was ≈2.4 N m^−1^ during the peeling adhesion test, comparable to that of clinically used fibrin glue. (Figure S2L, Supporting Information).

To probe the electrochemical properties of GMP and GMPE hydrogels, a hydrogel electrode was prepared by in situ gelation of electroconductive hydrogels onto a piece of indium‐tin oxide (ITO). cyclic voltammetry (CV), and electrochemical impedance (EIS) were performed with 0.1 m phosphate buffered saline (PBS, pH 7.4) as the electrolyte. Compared to the GM hydrogel, the GMP and GMPE hydrogels showed significantly improved anodic and cathodic currents (Figure 1D). The CV curves showed similar oxidation and reduction current values for GMP and GMPE hydrogels. The EIS imaging showed a quasi‐semicircle in the high‐frequency region of the Nyquist plots of the GMP and GMPE hydrogels, indicating that they exhibited good redox activity. Additionally, the diameter of this quasi‐semicircle was related to the charge transfer resistance. The larger the radius of the circle, the larger the charge transfer resistance. The diameter of the semicircle for the GM hydrogel was significantly larger than that for the GMPE and GMP hydrogels, which indicated that the GMP and GMPE hydrogels both exhibited better electrical performance in comparison with GM hydrogels. In addition, the current–voltage (*I*–*V*) curves showed that the conductivities of GMP and GMPE hydrogels were 1.83 × 10^−3^ and 1.49 × 10^−3^ S cm^−1^, respectively, which were significantly higher than those of the GM hydrogel. As the Bode plots show, both GMP and GMPE hydrogels showed significantly low impedance values that were between 300 Hz and 1 kHz compared with GM hydrogel. These values are within the frequency of the exchange signals observed in nerve cells.^[^ ^27^ ^]^ Together, these data show that GMP and GMPE hydrogels exhibited similar electrical properties to each other, indicating that the introduction of exosomes had no obvious effect on the electrical properties of the electroconductive hydrogels. An isolated spinal cord circuit test was used to further evaluate the ability of the hydrogel to transmit electrical signals. After the ex vivo mouse spinal cord was transected, no electrical signal transmission was recorded below the injury site (Figure 1E). When two ends of injury site were bridged by GMPE hydrogel, the stimulating electrical signals were able to be transmitted (Figure 1E), which indicated that the GPME hydrogel could partly restore endogenous electrical signaling transmission. In addition, the GMPE hydrogel also exhibited electrical stability in physiological medium for more than 2 weeks (Figure S2M, Supporting Information), which shows that it has potential property for long‐term in vivo use.

The retention of BMSC‐exosomes in GMPE hydrogel were investigated to evaluate the delivery capacity of the hydrogels. To visualize exosomes loaded on hydrogel, exosomes was labeled by PKH26 dyes and imaged via confocal microscopy. The 3D immunofluorescence (IF) imaging showed that in addition to a small amount of exosomes aggregation, most of the exosomes were evenly distributed on the surface of the GMPE hydrogel (Figure 1F). The control group with PKH26 dye applied to the blank solution without exosomes has no fluorescent signal, indicating that there are no residual dye aggregates in the PKH26 dyes‐labeled exosomes (Figure S2N, Supporting Information). After the exosomes were immobilized in the hydrogel, the GMPE hydrogels were stored in PBS at 4 °C, and the retention of exosomes loaded in hydrogel was assessed over time with laser scanning confocal microscopy (Figure S3A, Supporting Information). These results revealed that exosomes retention times for the GMPE hydrogel were up to 14 days (Figure S3B, Supporting Information) and, with more than 90% of the exosomes exhibiting unobstructed release (Figure S3C, Supporting Information). The release of loaded exosomes from the GMPE hydrogel was also confirmed for up to 14 days, at which time exosomes could fully exert their immune‐modulating and neuroregeneration‐enhancing effects during the early stages after injury. As shown in Figure S3B,C of the Supporting Information (also shown below), about 80% of exosomes were released from the GMPE hydrogel without TA immediately after it was immersed into the culture medium. In addition, the exosomes retention times for the GMPE hydrogel without TA were only up to 7 days. These results confirm the existence of reversible interactions between exosomes and hydrogels. Furthermore, a review of some literature shows that TA can interacts with biomacromolecules, including DNA and proteins, such as thrombin, gelatin, collagen, and mucin, via reversible hydrogen bonds.^[^ ^26^, ^28^, ^29^ ^]^ BMSC‐exosomes were immobilized in the TA‐doped GMP hydrogel may due to the reversible hydrogen bond formation. In vivo live imaging of PKH26 labeled exosomes showed they loaded within the hydrogel and remain at the injury site for 5 days after implantation, but the exosomes delivered in PBS were almost undetectable at the injury site (Figure S3D, Supporting Information). Moreover, the histological test further confirmed that the exosomes were phagocytosed by endogenous cells in vivo (Figure S3E, Supporting Information). These results indicated that hydrogels prolonged the residence time of exosomes and extended their release in the injury area.

To predict the possible mechanism of BMSC‐exosomes on axonal regeneration, neural differentiation and anti‐inflammatory function, the expression levels of related microRNAs (miRNAs) in exosomes were measured. The GMPE BMSC‐exosomes highly expressed axonal regeneration‐related miRNAs including miR29a, miR21, miR29c, miR26a, miR20a, miR9‐5p, miR182, miR128, miR133b, miR431, and miR124; remyelination‐related miRNAs, including miR145a, miR9‐5p, miR148b, miR138, miR338, miR219a, miR1a, and anti‐inflammatory‐related miRNAs, including miR199a, miR99a, miR146a, miR181a, and miR411. Among them, miR29a, miR21, miR29c, miR26a, miR145a, and miR199a were relatively high in BMSC‐exosomes (Figure 1G). We have retrieved, extracted and analyzed transcriptomic miRNAs expression in BMSC‐derived exosomes from the Gene Expression Omnibus (GEO) database. Consistent with our PCR results, the heatmap showed that the BMSC‐derived exosomes contained higher concentrations of anti‐inflammatory‐related, remyelination‐related, and axonal regeneration‐related miRNAs (Figure S4A,B, Supporting Information). Moreover, PKH26‐labeled exosomes were clearly detected in the cytoplasm of BV‐2 microglial cell (BV2 cell), suggesting successful in vitro endocytosis of exosomes released from the GMPE hydrogel (Figure 1H). The level of the anti‐inflammatory‐related miRNAs in BV2 was significantly higher after endocytosis of BMSC‐exosomes (Figure 1I), especially, miR199a levels were nearly threefold higher in BV2 cells (Figure 1I). NSCs can also normally phagocytize exosomes released from the GMPE hydrogel and the axonal regeneration‐related and remyelination‐related miRNAs in NSCs significantly upregulated after endocytosis of exosomes (Figure 1J,K). In particular, miR21 was upregulated fourfold and miR145a was upregulated threefold in NSCs (Figure 1K).

### 2.4 Biocompatibility and Biodegradability of the Hydrogels

The viability of cultured cells was tested using live/dead staining and a cell counting kit‐8 (CCK‐8) assay. The density of dead NSCs on the GMP hydrogel was significantly higher than that on the GM hydrogel (Figure S5A, Supporting Information). However, after exosomes were immobilized into the GMP hydrogel, the cell viability in the GMPE hydrogel cultures significantly increased, even more than that of the GM hydrogel group, as indicated by the live/dead staining and CCK‐8 assay results (Figure S5A,B, Supporting Information). Cytoskeleton staining was performed 3 days after cell were cultured in each condition (Figure S5C, Supporting Information). NSCs adhered to the plate surface and all three hydrogels. The spreading area and synaptic length of the cultured cells were significantly more on the GMPE hydrogel when compared to the other three groups (Figure S5D, Supporting Information). These results demonstrated that exosomes could function as biologically active microparticles that increased the in vitro cytocompatibility of the electroconductive hydrogels.

Subcutaneous implantation of the hydrogels was performed in mice to test their in vivo degradation. Three weeks after implantation, the GM hydrogel almost completely degraded, while the GMPE and GMP hydrogels were still detected 6 weeks after implantation (Figure S6A, Supporting Information). These results indicated that the degradation rate of the GMPE and GMP hydrogels was significantly lower than that of the GM hydrogel. However, the volume of the GMP and GMPE hydrogels in both the diameter and thickness was reduced 6 weeks after implantation when compared to their preimplantation volumes. In particular, the border of the electroconductive hydrogels became less defined (Figure S6A, green arrow, Supporting Information), suggesting that the electroconductive hydrogels degraded to a certain extent within 6 weeks. Hematoxylin–eosin (HE) staining also showed that a small amount of mononuclear inflammatory cells invaded the GMP and GMPE hydrogels, which corresponded with the degraded interface between the tissue and the electroconductive hydrogels 6 weeks after hydrogel implantation (Figure S6B, Supporting Information). Additionally, histological staining revealed that the PPy chain broke into PPy nanoparticles and that the cytoplasm of monocytes contained a large number of PPy nanoparticles, which suggested that the endocytosis of PPy nanoparticles may contribute to PPy degradation.

On the other hand, the histological staining analysis of the hydrogel implantation site also revealed the formation of a fibrous capsule around the GMP and GMPE hydrogels 1 week after hydrogel implantation, indicating collagen deposition, which is a part of a normal inflammatory response (Figure S6B,C, Supporting Information). However, the fibrotic capsule of the GMPE hydrogel was significantly thinner than that of the GMP hydrogel (Figure S6C, Supporting Information). Moreover, the density of invasive mononuclear inflammatory cells significantly decreased 6 weeks after implantation, especially in the GMPE hydrogel group (Figure S6D, Supporting Information). These results demonstrated that exosomes decreased the inflammatory responses initially caused by the electroconductive hydrogels. In addition, the histological analysis confirmed that there was no obvious accumulation of hydrogel degradation products and no noticeable pathological abnormalities in the major organs (i.e., heart, liver, spleen, lung, and kidney) in the mice treated with each hydrogel when compared with the control group (Figure S7A, Supporting Information). Moreover, there were no significant variations in the levels of alanine aminotransferase (ALT), aspartate aminotransferase (AST), and total protein (TP) in the GM, GMP, and GMPE hydrogel groups compared to the sham group, suggesting the hydrogels did not cause systemic toxicity (Figure S7B, Supporting Information). The in vitro hemolytic property is a standard method of assessing the hemocompatibility of biomaterials. Serum extracted from whole blood and hydrogel cocultures had a clear yellow color similar to that of the PBS control group, but the Triton‐100X group was bright red in color (Figure S7C, Supporting Information). The optical density value of serum from three hydrogel groups was also similar to PBS group and that of all groups was significantly lower than that of the Triton‐100X group (Figure S7D, Supporting Information). The hemolysis ratios of the three groups were all below 1%, which indicated that the GMPE hydrogels had excellent hemocompatibility as nerve repair materials.

### 2.5 GMPE Hydrogel Promotes M2 Microglial Polarization

**Figure 2:**
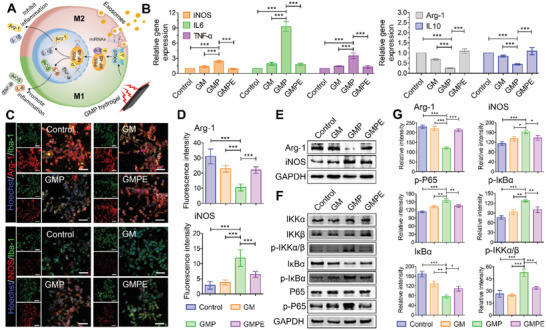
GMPE hydrogels promote microglia M1 to M2 switch by activating the NF‐*κ*B pathway. A) An illustration of microglia switching from an M1‐ to M2‐dominant phenotype through NF‐*κ*B pathway activity. B) RT‐qPCR results of the level gene expression of anti‐inflammatory factors Arg‐1 and IL‐10 and the proinflammatory factors iNOS, IL‐6, and TNF‐*α* in BV2 cells cultured on hydrogels (*n* = 3). C) IF imaging showing the proportion of Arg‐1 positive and iNOS positive BV2 cells cultured in each hydrogel treatment group. Green IF represents the microglia/macrophage specific protein marker Iba‐1, red fluorescence represents the M1/M2 microglia/macrophage phenotype marker iNOS or Arg‐1, blue fluorescence represents the nuclear marker Hoechst 33342. Scale bars: 200 µm. D) Quantification of fluorescence intensity of iNOS and Arg‐1 level in each hydrogel treatment group (*n* = 5). E) GMPE hydrogel promoted BV2 cell M2 polarization. F) GMPE hydrogel regulated the expression of the relative proteins of NF‐*κ*B pathway, further indicating that the GMPE hydrogel promotes BV2 cell M2 polarization through NF‐*κ*B pathway activation. G) Protein band intensity was quantified using ImageJ (*n* = 3). Statistical differences were determined using an ANOVA with Bonferroni's multiple comparison test (* *p* < 0.05, ** *p* < 0.01, *** *p* < 0.001).

To assess whether the GMPE hydrogel indeed acted as an anti‐inflammatory agent, we cocultured the GMPE hydrogel with a mouse microglia BV2 cell line. Microglia with specific markers can be divided into proinflammatory M1 or anti‐inflammatory M2 phenotypes (**Figure** **2**A). The M1 markers mainly include the protein inducible nitric oxide synthase (iNOS), interleukin (IL)‐6, and tumor necrosis factor alpha (TNF‐*α*), while M2 markers include arginase‐1 (Arg‐1) and IL‐10.^[^ ^30^ ^]^ Gene expression showed that mRNA levels of the anti‐inflammatory cytokines Arg‐1 and IL10 were significantly higher in the GMPE group than in the GMP group. The mRNA expression levels of the proinflammatory cytokines iNOS, IL‐6, and TNF‐*α* in BV2 cells cultured on GMPE hydrogel were significantly lower than those in cells cultured on the GMP hydrogel (Figure 2B). IF imaging of the cell cultures showed that the number of iNOS‐positive cells was significantly higher, while the number of Arg‐1‐positive cells was significantly lower in the GMP hydrogel than in the control and GM groups (Figure 2C,D). After exosomes were introduced into the electroconductive hydrogels, the number of iNOS‐positive cells significantly decreased and the number of Arg‐1‐positive cells was significantly increased. Inflammation regulation, including iNOS and Arg‐1 protein expression, was evaluated by western blotting (WB) analysis, which demonstrated that exosomes inhibited the inflammation caused by the electroconductive hydrogels (Figure 2E,G). To investigate the possible mechanism by which the GMPE hydrogel repopulated microglia, we further assessed the relative levels of proteins in the inflammatory NF‐*κ*B pathway (Figure 2F,G). The GMPE hydrogel treatment inhibited the NF‐*κ*B pathway, indicated by the decreased expression of phosphorylated‐IKK*α*/*β* (p‐IKK*α*/*β*), p‐I*κ*B*α*, and p‐P65 (Figure 2F). Expect for more I*κ*B*α* consumed, the total I*κ*B*α* level in the GMP hydrogel was significantly lower than that in the GMPE hydrogel, while the total amounts of IKK*α*, IKK*β*, P65 proteins were not reduced (Figure 2F,G). The phosphorylation of I*κ*B*α* protein was irreversibly inhibited by BAY 11–7082, and significantly reduced the level of p‐I*κ*B*α* (Figure S8A, Supporting Information). Additionally, p‐P65 protein expression in BV2 cells cultured on electroconductive hydrogels was significantly lower than that in cells cultured without inhibitors (Figure S8B,C, Supporting Information). Furthermore, the level of Arg‐1 protein was significantly higher, while that of iNOS protein was significantly lower in the BAY 11–7082 inhibition group compared to that in the samples that were not treated with BAY 11–7082. These results demonstrated that exosomes promoted microglia M2 polarization may through NF‐*κ*B pathway.

### 2.6 NSCs Differentiation on Hydrogels

**Figure 3:**
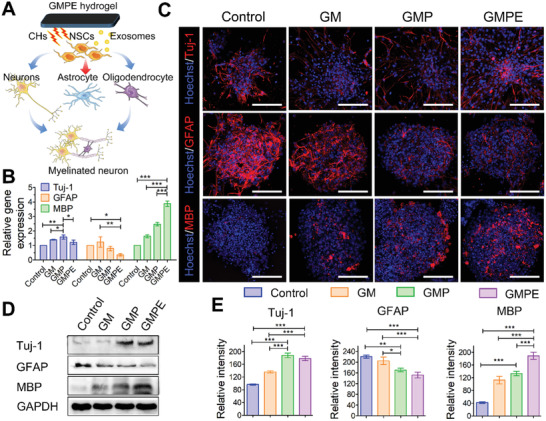
NSCs differentiation on hydrogels. A) An illustration showing that the GMPE hydrogel promotes NSC neuron and oligodendrocyte differentiation to form myelinated neurons, but inhibited astrocyte differentiation. B) The RT‐qPCR data show GMPE promote MBP expression (*n* = 3). C) IF imaging of NSC neuron, astrocyte, and oligodendrocyte differentiation after 7 days on control or hydrogel conditions. Red IF represents the neuron marker Tuj‐1, astrocyte marker GFAP, or oligodendrocyte marker MBP, respectively. Scale bars: 100 µm. D) WB analysis detected Tuj‐1, GFAP, and MBP protein expression in NSCs cultured on hydrogels and control conditions for 7 days. E) Protein band intensity was quantified (*n* = 3). Statistical differences were determined using an ANOVA with Bonferroni's multiple comparison test (* *p* < 0.05, ** *p* < 0.01, *** *p* < 0.001).

NSCs are multipotent, self‐renewing cells with the potential to differentiate into three cell sublineages, astrocytes, oligodendrocytes, and neurons (**Figure** **3**A).^[^ ^31^ ^]^ Compared to the control and GM groups, both GMP and GMPE hydrogels promoted the gene expression of the neuronal differentiation marker *β*3‐tubulin (Tuj‐1) and the oligodendrocyte differentiation marker myelin basic protein (MBP); however, the expression of the astrocyte marker glial fibrillary acidic protein (GFAP) gene was inhibited in these culture conditions (Figure 3B). Furthermore, NSCs cultured on the GMPE hydrogel expressed the higher levels of MBP and the lower levels of GFAP in comparison to the GMP hydrogel culture conditions, although the expression of GFAP showed no significant difference between the GMPE and GMP groups. The IF image and WB analysis showed that the protein expression for MBP and GFAP was consistent with their gene expression (Figure 3C,E). These results indicated that both electroconductive hydrogels can promote NSC neuronal and oligodendrocyte differentiation, while inhibit the astrocyte differentiation. The additional exosomes on the GMPE hydrogel further promoted oligodendrocyte differentiation in NSCs when compared to GMP hydrogel.

### 2.7 Axon Outgrowth in Differentiated NSCs and Dorsal Root Ganglion (DRGs) Grown on Hydrogels

**Figure 4:**
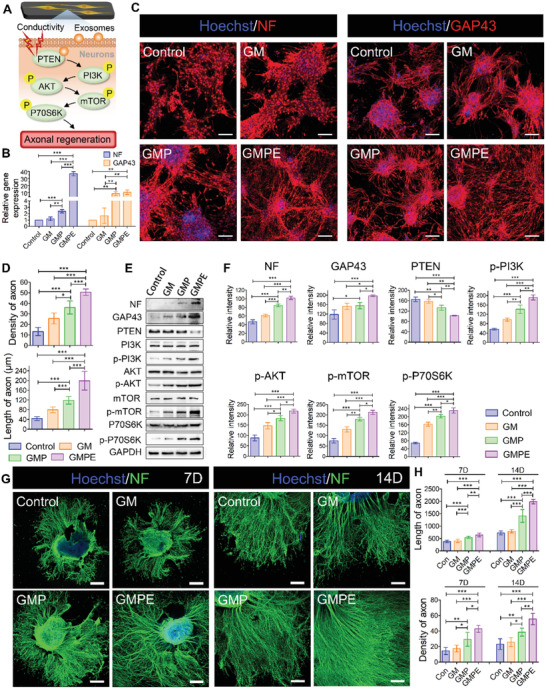
Axon outgrowth on hydrogels. A) Illustration of the BMSC‐exosomes electroconductive hydrogel mechanism and synergistic promotion of axon outgrowth through the activation of the PTEN/PI3K/AKT/mTOR pathway. B) RT‐qPCR indicating that GMPE hydrogels can promote NF and GAP43 gene expression (*n* = 3). C) IF images of the axon‐associated proteins NF and GAP43 in NSCs grown on hydrogels for 7 days. Red IF represents the NF or GAP43, respectively. Scale bars: 100 µm. D) The density (*n* = 5) and length (*n* = 11) of axons were quantified using ImageJ software. E) WB result of the expression of NF and GAP43 proteins and the relative protein expression of the PTEN/PI3K/AKT/mTOR pathway in NSCs cultured on control or hydrogel conditions for 7 days. F) Protein band intensity was quantified (*n* = 3). G) IF images of the NF positive axon in DRGs grown on hydrogels for 7 days. H) The density (*n* = 5) and length (*n* = 11) of axons were quantified using ImageJ software. Statistical differences were determined using an ANOVA with Bonferroni's multiple comparison test (* *p* < 0.05, ** *p* < 0.01, *** *p* < 0.001).

The expression of the axon‐associated proteins neurofilament (NF) and growth associated protein‐43 (GAP43) was used to investigate the effect of hydrogels on regenerative axon growth (**Figure** **4**A). The NF gene levels in the GMPE group were more than 30‐fold higher than those in the control and GM groups and were more than 10‐fold higher than those in the GMP group (Figure 4B). Although the GAP43 level was higher in the GMPE groups than in the GMP group, the difference between the two groups was not significant. However, GAP43 expression levels in the GMPE group were significantly higher than those in the GM and control groups. Axon outgrowth from NF/GAP43‐positive cells and the formation of a neural synaptic network was observed after 7 days in NSCs cultured on GMPE hydrogel (Figure 4C). The axonal density in the GMPE hydrogel group was the highest, with an axonal length of 199.23 ± 38.53 µm, which was relatively longer than that in the GMP (118.82 ± 15.76 µm), GM (79.64 ± 12.14 µm), and control (43.79 ± 7.95 µm) groups (Figure 4D). The protein levels of NF and the GAP43 postsynaptic marker were also increased in the GMPE group, which consistent with gene expression and protein localization data (Figure 4E,F). Together, these results indicated that the GMPE hydrogel promoted axon outgrowth and neural synaptic network formation. To investigate the possible mechanism of axon extension, we measured the relative protein expression of the phosphatase and tension homolog gene on chromosome 10 (PTEN)/phosphatidylinositol 3‐kinase (PI3K)/protein kinase B (AKT)/mammalian targets of the rapamycin (mTOR) pathway (Figure 4E,F). A significant decrease in PTEN was observed in the GMPE group compared with the other three groups. Additionally, although the expression levels of phosphorylated (i.e., active) PI3K (p‐PI3K), p‐AKT, p‐mTOR, and p‐P70S6K were significantly upregulated in the GMPE group, the total amount of these proteins was not different between the tested growth conditions. The p‐PI3K, p‐AKT, p‐mTOR, and p‐P70S6K expression levels were also higher, while PTEN expression was lower in the GMP hydrogel than in the GM and control groups. These data validated our hypothesis that electroconductive hydrogels with exosomes promoted axon spread via the costimulation of the PTEN/PI3K/AKT/mTOR pathway. To further verify our hypothesis, p‐mTOR expression was downregulated using m‐TOR siRNA (Sim‐TOR) and rapamycin (Rp) after NSCs cultured on the GMPE hydrogel. The siRNA directly binds the target mRNA, while Rp inhibits target protein phosphorylation, which both lead to a significant decrease in the p‐mTOR protein levels (Figure S9A, Supporting Information). Rp was dissolved in dimethyl sulfoxide (DMSO); therefore, we included a DMSO group as a control and found that DMSO had no effect on the experimental outcome. The siRNA#1 construct had the greatest effect on the target gene compared to the other clones; therefore, it was used for further experiments (Figure S9B, Supporting Information). When the PTEN/PI3K/AKT/mTOR pathway was downregulated, the protein expression levels of both NF and GAP43 decreased in the siRNA and Rp groups (Figure S9C,D, Supporting Information). Protein quantification and localization demonstrated that both axonal density and length of the spread from the neuron differentiated from NSCs decreased when the pathway was inhibited (Figure S9E–F, Supporting Information). These findings further demonstrated that GMPE hydrogel promoted axon extension possible by activating PTEN/PI3K/AKT/mTOR pathway.

Compared to NSCs, peripheral neurons possess a stronger intrinsic potentiality for axonal regeneration and are conventionally used in axonal regeneration research. To test their responsiveness toward GMPE hydrogel, we harvested the dissociated DRGs to culture on the different hydrogels. As IF images showed, the length and density of axons on the GMPE group were significantly increased than those in control, GM and GMP group at 7 days or 14 days (Figure 4G). Quantitative analysis of neurite outgrowth showed that the axonal length was 1985.27 ± 88.24 µm in the GMPE group, which was relatively longer than that in the GMP (1409.09 ± 255.97 µm), GM (791.55 ± 64.60 µm), and control (723.09 ± 78.96 µm) groups (Figure 4H). The axonal density in the GMPE hydrogel group was also 1.54 ± 0.34, 2.08 ± 0.71, and 2.08 ± 0.66 fold higher than that in the GMP group, GM group, and control group, respectively (Figure 4H). These results further demonstrated that the GMPE hydrogel promoted axon outgrowth.

### 2.8 The GMPE Hydrogel Improved Mouse Pathology and Motor Function after SCI

**Figure 5:**
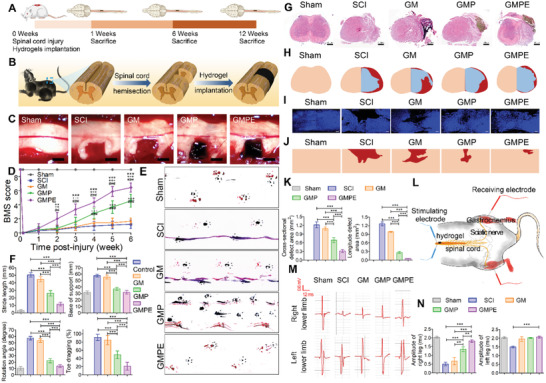
Functional recovery of mice in different groups. A) The experimental timeline schematics. B) Illustration of the spinal cord hemisection and hydrogel implantation. C) Different hydrogels were implanted at the cavitary site. Scale bars: 1 mm. D) Right hindlimb locomotor recovery in mice was evaluated using BMS scoring on a grid. Mice treated with the GMPE hydrogel had improved BMS locomotor scores 2 weeks after injury (*n* = 9) when compared to GMP (* *p* < 0.05, ** *p* < 0.01, *** *p* < 0.001) and GM (+ *p* < 0.05, ++ *p* < 0.01, +++ *p* <0.001) hydrogels, and SCI (# *p* < 0.05, ## *p* < 0.01, ### *p* <0.001). E) Representative footprints used to analyze recovery of hindlimb motor function. The forelimb footprints are shown in blue, and the hindlimb footprints in red. F) Stride length, base of support, rotation angle, and toe dragging were used to quantify the recovery of locomotion at 6 weeks after injury (*n* = 9). G) HE staining showing the morphology of transverse spinal cord sections of sham and hydrogel implantation after SCI. Scale bars: 500 µm. H) Representative reconstructions of the transverse spinal cord sections. Flesh‐colored areas represent normal tissue, cavitary areas are highlighted in red, and blue‐colored areas represent regenerated tissue. I) IF images illustrating the morphology of longitudinal sections of the spinal cords. Scale bars: 200 µm. J) Representative images of reconstructed spinal cord longitudinal section. Flesh‐colored areas represent normal tissue, while red‐colored areas represent the cavitary areas. K) Cavity volume of transverse spinal cord and longitudinal sections was quantified (*n* = 3). L) Illustration of the CMAPs testing protocol in mice from GMPE hydrogel treatment group. M) CMAPs results from normal mice and those in different hydrogel implantation groups 6 weeks post‐treatment. N) Quantification of the CMAPs amplitudes measured in mice from sham and hydrogel treatment groups (*n* = 3). Statistical differences were determined using an ANOVA with Bonferroni's multiple comparison test (* *p* < 0.05, ** *p* < 0.01, *** *p* < 0.001).

The experimental timeline schematic was shown in **Figure** **5**A. The Basso mouse scale (BMS) score was used to evaluate mouse functional recovery after SCI. Each mouse exhibited normal locomotor activity of the right hindlimbs before injury (9 score). Surgical procedure was shown in Figure 5B,C. Immediately after right spinal cord hemisection, the animals exhibited complete paralysis (0 score) of the right hindlimbs (Figure 5D). The right hindlimb BMS scores were no more than 2 in the SCI and GM groups 6 weeks after SCI, indicating a limited capacity for organism self‐healing. By contrast, statistically significant locomotor functional recovery was observed in the GMPE groups in the 2 weeks postoperation, suggesting that the GMPE hydrogel promoted functional recovery at an early stage after SCI. In the GMP hydrogel group, the locomotor function was similar to that in the SCI and GM hydrogel groups 2 weeks postinjury, but significantly improved 4 weeks after SCI, although this improvement was significantly lower than that in the GMPE hydrogel. These findings showed that electroconductive hydrogels could partially promote mouse movement recovery. Six weeks postoperation, most GMPE hydrogel‐implanted mice presented normal weight support (7 score), while most mice treated with the GMP hydrogel exhibited plantar placement with support (5 score) and mice in the SCI and GM groups exhibited extensive ankle movement (1–2 score). These results strongly suggest that the combination of electroconductive hydrogels and exosomes significantly improved functional recovery after SCI.

The footprint analysis results were consistent with the BMS scores and are summarized in Figure 5E. Mice treated with the GMP hydrogel exhibited plantar placement with support but stepped without coordination; however, mice treated with the GMPE hydrogel exhibited coordinated crawling using their front and rear limbs, while mice still dragged their right hindlimbs in the SCI group. Six weeks after SCI, toe dragging and base of support were significantly reduced in the GMPE groups, suggesting these mice had restored weight bearing in comparison with those in the SCI, GM, and GMP groups (Figure 5F. Furthermore, neither mean stride length nor the rotation angle improved in the GMPE group when compared with the other three groups, indicating hydrogel implantation improved coordination between the fore‐ and hind‐paws. Footprint analysis further demonstrated that electroconductive hydrogels with exosomes enhanced coordination between the fore‐ and hind‐paws.

Conventional magnetic resonance imaging (MRI) is routinely used for qualitative assessment of the injured spinal cord pathology (Figure S10A, Supporting Information). Mice in the sham group showed normal spinal cord morphology, while those in the SCI and GM groups, the cross‐sectional areas at the injury site of the spinal cord showed that the normal pathology of the spinal cord could not be seen 6 weeks after injury (Figure S10A, Supporting Information). Similarly, right hemisection spinal cord defect normal morphology from the loss of normal nervous tissue at the injury site were observed in tissue HE staining (Figure 5G). In transverse spinal cord sections, the cavitary area was 1.23 ± 0.08 mm^2^ in SCI groups and was 1.08 ± 0.05 mm^2^ in GM groups (Figure 5H,K). By contrast, both the GMP and GPME hydrogels reduced vacuolation and the cavitary area in the GMPE group (0.68 ± 0.07 mm^2^) was significantly smaller than that in the GMP group (0.31 ± 0.07 mm^2^) 6 weeks after surgery. The longitudinal cavitary area was 0.05 ± 0.01 mm^2^ in GMPE group mice, which was significantly smaller than that of the SCI (1.27 ± 0.07 mm^2^), GM (0.99 ± 0.02 mm^2^), and GMP (0.27 ± 0.03 mm^2^) groups (Figure 5I,K). Also, the lesion volume was smallest in the GMPE group compared to the other groups at 3 and 6 weeks after SCI (Figure S10B, Supporting Information). These results confirmed that the GMPE hydrogel reduced the cavitary area and facilitating cell infiltration and tissue formation. Electrophysiological analyses were performed to estimate the degree of functional recovery. After the spinal cord above the injury site was provoked with a stimulating electrode, the activity of the target muscle was recorded by a receiving electrode, reflecting the functional recovery after SCI (Figure 5L). The change in compound muscle action potentials (CMAPs) signal amplitude was recorded from the gastrocnemius and analyzed individually. CMAPs signals from the mouse right hindlimbs in the SCI group decreased almost fivefold in comparison to those in the sham group 6 weeks post‐SCI (Figure 5M,N). By contrast, the CMAPs signal amplitude recorded from the GMPE group was 1.82 ± 0.06 mV, which was significantly greater than that in the GM (0.59 ± 0.19 mV) and SCI (0.45 ± 0.10 mV) groups (Figure 5M,N). Although the amplitude (1.36 ± 0.11 mV) in the GMP hydrogel group was significantly lower than that in the GMPE hydrogel, it was still greater than that in the SCI and GM groups. These data demonstrated that the GMPE hydrogel was superior to the GMP hydrogel in improving motor function after SCI.

### 2.9 Hydrogels Modulate Inflammation In Vivo

**Figure 6:**
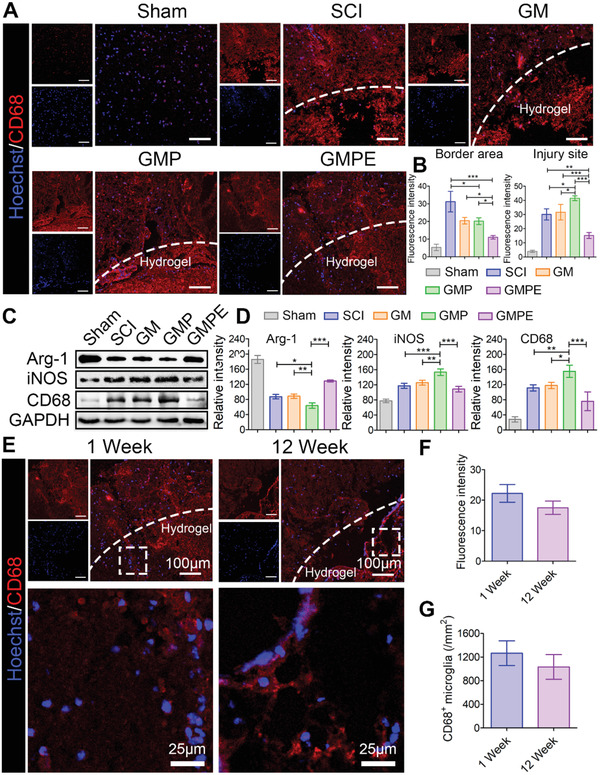
GMPE hydrogel suppressed early inflammation after SCI. A) The CD68‐positive cells were significantly decreased in the GMPE hydrogel groups compared to the other treatment and control SCI conditions. Red IF represents CD68. Scale bars: 100 µm. B) The intensity of CD68 fluorescence signals were quantified (*n* = 3). The intensity of CD68 was highest in the GMP group at the center of injury site when compared to the other treatments. C) WB of CD68, iNOS, and Arg‐1 protein expression in the different treatment groups. D) Graphs of the protein band intensity quantification (*n* = 3). E) The CD68‐positive cells were evaluated at 1 week and 12 weeks in the GMPE group. Red IF represents CD68. Quantification of F) CD68 fluorescence intensity (*n* = 3) and G) CD68^+^ microglia number (*n* = 3). Statistical differences were determined using an ANOVA with Bonferroni's multiple comparison test (* *p* < 0.05, ** *p* < 0.01, *** *p* < 0.001).

Inflammatory reactions usually lead to a series of secondary insults after SCI. Therefore, inhibition of inflammation at the early stage can provide a good environment for SCI repair. Seven days after SCI, the spinal cords of mice in each treatment group were harvested to evaluate the inflammation caused by the injury and materials. The density of CD68‐positive cells was significantly greater at the injury site in the SCI group than in the sham group, indicating that severe inflammation occurred early after SCI (**Figure** **6**A). The CD68 IF intensity increased in the GMP group at the center of the injury site and was higher than that in the SCI group (Figure 6B), which suggested that electroconductive hydrogels further aggravated local inflammation. Transplantation of the GMPE hydrogel significantly reduced the density of CD68‐positive cells and the expression levels of proinflammatory factors (iNOS), while increasing the expression levels of anti‐inflammatory factors (Arg‐1, Figure 6C,D). Also, the numbers of CD68^+^ cells in the injury site were determined at the 1‐ and 12‐week time points using ImageJ software. As Figure 6E,G showed, the CD68 fluorescence intensity and CD68^+^ cells in the GMPE group at 12 weeks were lower than that at 1 weeks, although this difference is not significant. Severe inflammatory reactions usually occur at the early stage of spinal cord injury, leading to a series of secondary insults. After an early, long‐term and chronic inflammation may promote ongoing tissue damage while simultaneously engaged in healing and repair. BMSC‐exosomes released from GPME hydrogel can inhibit inflammation at the early stage but cannot completely eliminate the long‐term chronic inflammation. However, BMSC‐exosomes can minimize the deleterious effects of inflammation at an early stage, providing a good environment to ensure long‐term neuronal regeneration. These results demonstrated the immunomodulatory properties of BMSC‐exosomes. This result was similar to that observed in subcutaneous implantations as previously reported demonstrated that implantation of the GMPE hydrogel could inhibit the inflammatory response at an early stage of SCI.

### 2.10 Hydrogels Enhanced NSCs Recruitment and Neural Regeneration In Vivo

**Figure 7:**
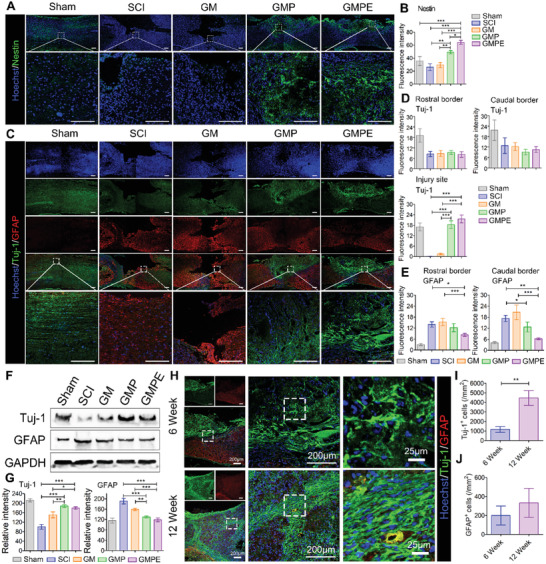
GMPE hydrogel implantation promotes endogenous NSCs recruitment and neuronal regeneration. A) Nestin‐positive endogenous NSCs spontaneously migrated into the GMPE hydrogel 6 weeks after SCI. Green IF represents the NSC marker nestin. Scale bars: 200 µm. B) Graph of the quantification of nestin IF intensity 6 weeks after SCI. The region of interest (ROI) was the injury site center (*n* = 3). C) The GMPE hydrogel promotes endogenous NSC neuronal regeneration while inhibiting astrocytic proliferation in vivo. Green IF represents the neuronal marker Tuj‐1. Red IF represents the astrocytic marker GFAP. Scale bars: 200 µm. D) Graph of the Tuj‐1 IF intensity quantification. The fluorescence intensity was measured at three ROIs: the rostral and caudal borders, and the injury site center (*n* = 3). E) Graph of GFAP IF intensity quantification at same timepoint at the ROIs: rostral and caudal borders of the injury site (*n* = 3). F) WB analysis of the neuronal and astrocytic iconic protein markers in each treatment group. G) Quantitative measurement of protein band intensity (*n* = 3). H) Neuronal regeneration induced by GMPE hydrogel was also evaluated at 6 weeks and 12 weeks. Quantification of I) Tuj‐1^+^ cells (*n* = 3) and J) GFAP^+^ cells number (*n* = 3). Statistical differences were determined using an ANOVA with Bonferroni's multiple comparison test (* *p* < 0.05, ** *p* < 0.01, *** *p* < 0.001).

To further investigate the histological changes and the mechanism underlying functional recovery of SCI after GMPE hydrogel treatment, protein expression, and localization was used to access local NSCs recruitment and neural regeneration, and astrological scar formation at the injury site. Six weeks after injury, numerous nestin‐positive NSCs appeared around the injury site in the GMP and GMPE groups (**Figure** **7**A,B), as well as migration of nestin‐positive NSCs into the injury site in the GMP and GMPE hydrogel groups. However, nestin‐positive NSCs in the SCI and GM hydrogels were almost undetectable at the lesion center. In the GMP and GMPE hydrogel groups, we also observed large numbers of Tuj‐1‐positive neurons at the center of the lesion area, while these cells were relatively less present in the GM hydrogel and SCI groups (Figure 7C,D). However, the intensity of Tuj‐1 positive neurons was not significantly different among the SCI, GM, GMP, and GMPE groups at the rostral and caudal borders. These findings suggest that electroconductive hydrogels promoted the invasion of neurons into the hydrogels. Moreover, the number of GFAP‐positive astrocytes significantly increased at the caudal and rostral regions and formed an astrological scar around the lesion site in GM and SCI groups, which was more GFAP‐positive astrocytes than that in the GMP and GMPE hydrogel groups (Figure 7C,E). Thus, GMP and GMPE hydrogels inhibited GFAP‐positive astrocytic scar formation, providing a favorable microenvironment for later axonal regeneration. WB analysis was consistent with the IF results; the expression of Tuj‐1 in the GMPE group was similar to that in the GMP hydrogel but was much higher than that in the SCI and GM groups, while GFAP at the lesion site in the GMPE group was also similar to that in the GMP group and significantly lower than that in the GM and SCI groups (Figure 7F,G). Together, these results show that the GMPE hydrogel promoted endogenous NSCs recruitment, enhanced neuronal regeneration and inhibited astrocytic proliferation. In addition, the Tuj‐1‐positive cells in the GMPE group at 12 weeks were significantly higher than that at 6 weeks (Figure 7H–J), indicating that the GMPE hydrogel has the function of continuously inducing neural regeneration.

### 2.11 Axonal Regeneration and Remyelination In Vivo

**Figure 8:**
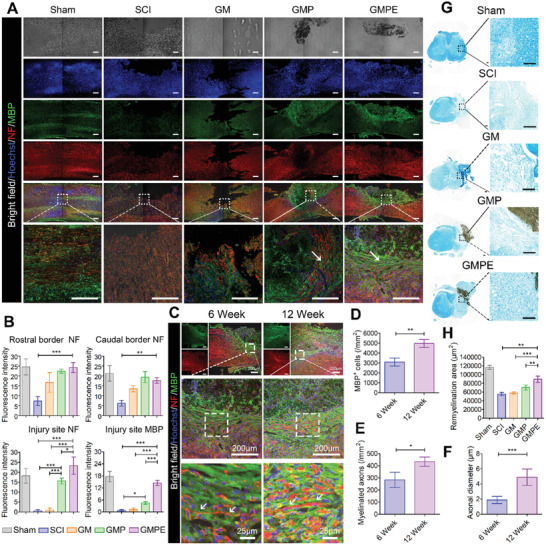
GMPE hydrogel implantation promotes axonal regeneration and remyelination in vivo. A) Axonal regeneration and remyelination were evaluated in the five treatment groups 6 weeks postoperation using IF imaging. Green IF represents the MBP oligodendrocyte marker. Red IF represents the NF axon marker. Scale bars: 200 µm. B) Graph of NF and MBP IF intensity quantified from ROIs at both ends and the center of the injury site (*n* = 3). GFAP IF intensity ROI was measured from the center of the injury site (*n* = 3). C) Myelinated axons regeneration was evaluated in the GMPE groups at the 6‐ and 12‐week time points. Green IF represents the MBP oligodendrocyte marker. Red IF represents the NF axonal marker. Quantification of D) MBP^+^ cells number (*n* = 3), E) myelinated axons number (*n* = 3), and F) axonal diameter (*n* = 5). G) Remyelination was evaluated by LFB staining, which showed that nerve myelin was detected around the GMPE hydrogel. Scale bars: 200 µm. H) Quantification of remyelination area (*n* = 3). Statistical differences were determined using an ANOVA with Bonferroni's multiple comparison test (* *p* < 0.05, ** *p* < 0.01, ***p < 0.001).

**Figure 9:**
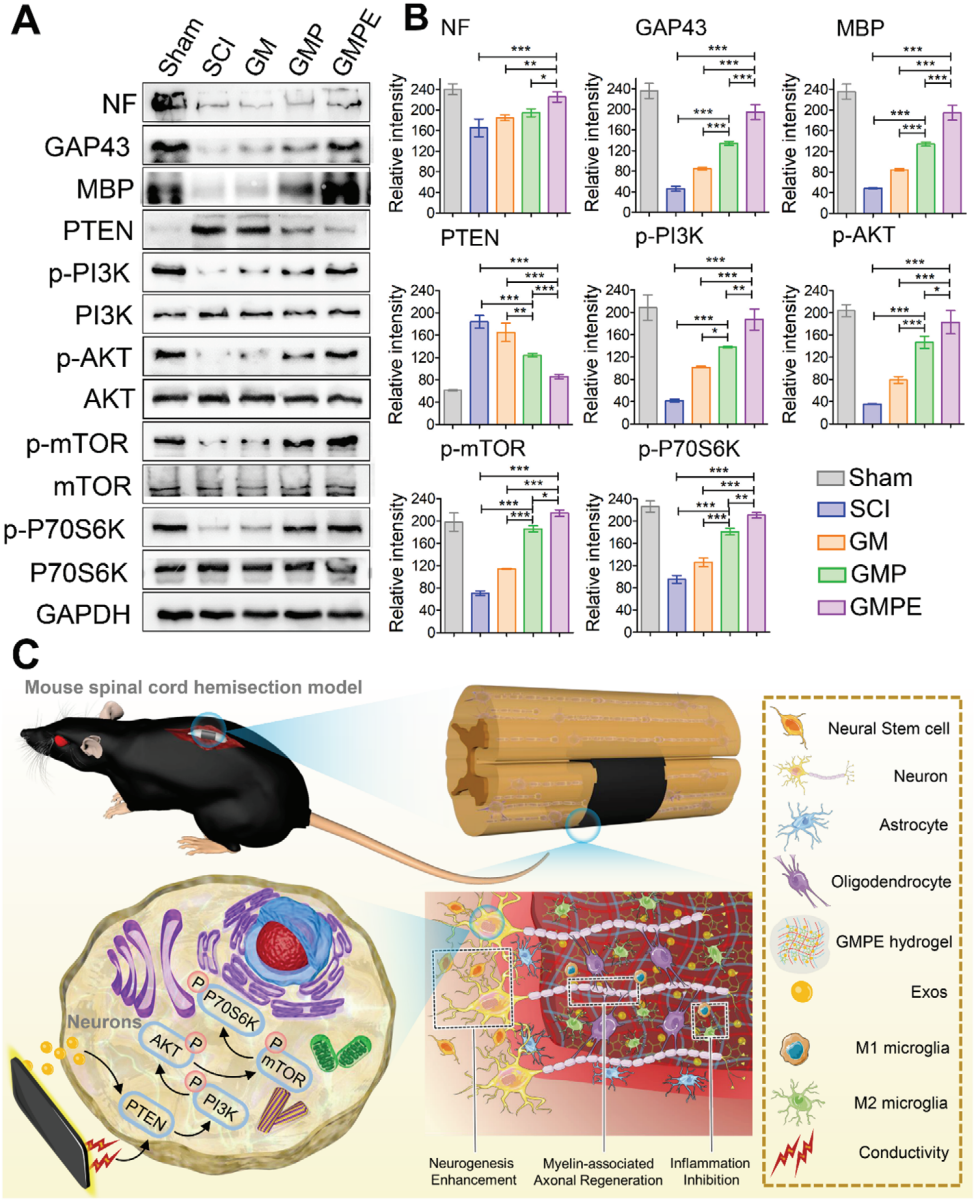
The mechanism of GMPE hydrogel promoting SCI repair. A) Protein expression of NF, GAP43, MBP, and PTEN/PI3K/AKT/mTOR pathway proteins. GADPH was included as an expression control. B) Graph of protein band intensity quantification (*n* = 3). C) Exosomes‐loaded electroconductive hydrogels synergistically enhance neuronal and oligodendrocyte differentiation of neural stem cells (NSCs) while inhibiting astrocyte differentiation, and also increase axon outgrowth via the PTEN/PI3K/AKT/mTOR pathway. Statistical differences were determined using an ANOVA with Bonferroni's multiple comparison test (* *p* < 0.05, ** *p* < 0.01, *** *p* < 0.001).

Axonal regeneration was quantified by determining the NF‐positive axons that regenerated into the lesion site. Longitudinally oriented NF‐positive fibers that originated from neuronal cells were observed in both the GMP and GMPE groups (**Figure** **8**A). However, these axons were wrapped around the material and appeared disordered in the GMP hydrogel group, (Figure 8A white arrows). The NF positive axons formed tended to connect both ends of the injury site in the GMPE group, while the axonal density between the GMP and GMPE hydrogel groups was similar at the rostral/caudal borders. However, when we compared axonal density at the injury site, it was highest in the GMPE group, relatively low in the GMP hydrogel treatment, and much lower in the SCI and GM groups (Figure 8B). These results demonstrate that GMPE hydrogel transplantation can induce axonal regeneration at the injury site. the MBP‐positive myelin sheath density was highest in the GMPE hydrogel group at the center of the injury site when compared to the SCI, GM, and GMP groups (Figure 8B). Furthermore, the regenerated axons in GMPE groups showed typical wrapping with myelin sheets (Figure 8C as white arrows indicated). In striking contrast, nearly no myelin‐associated axons were detected at the caudal ends or at the lesion site center of the SCI, and fewer disordered myelin‐associated axons were detected in the GM groups. Meanwhile, the number of MBP‐positive cells and myelinated axons in GMPE hydrogel at 12 weeks were both significantly higher than that at 6 weeks (Figure 8C,E). In addition, the axonal diameter in GMPE hydrogel at 12 weeks was 2.63 ± 0.36 fold higher than that at 6 weeks (Figure 8F). Luxol fast blue (LFB) staining showed that a large number of nerve myelin was detected around the GMPE hydrogel, while the density and the positive area of myelin were significantly lower in the other three groups (Figure 8G,H). These results strongly suggest that the GMPE hydrogel can also continuously promote myelinated nerve fibers regeneration. WB analysis was consistent with the protein localization results, indicating that NF and GAP43 were expressed more at the injury site in the GMPE group that in the GMP, GM, and SCI groups (**Figure** **9**A,B). Consistent with our in vitro experiments, the relative phosphorylation of PTEN/PI3K/AKT/mTOR pathway proteins was highest in the GMPE group, and the protein expression levels in the GMP group were significantly upregulated when compared to those in the SCI and GM groups (Figure 9A,B). These results further demonstrated that these axonal regeneration phenomena were associated with PTEN/PI3K/AKT/mTOR pathway activation (Figure 9C).

## 3 Discussion

Neuroinflammation, glial scar formation, and difficulty in axonal regeneration are the factors limiting regeneration capability after SCI.^[^ ^32^ ^]^ Scaffold biomaterial‐based treatment has been proposed as an appropriate axonal guidance approach to promote neural tissue regeneration.^[^ ^7^, ^18^ ^]^ The capacity to conduct electrical signals is an important requirement for the biomaterials used in neuroregeneration.^[^ ^33^ ^]^ Our electroconductive hydrogels exhibited suitable swelling and mechanical properties, and excellent electrical conductivity that were similar to native neural tissue, making them promising candidates for nerve injury repair.^[^ ^10^, ^33^ ^]^ The swelling ratio of the GMP and GMPE hydrogels was slightly reduced after PPy chain polymerized into the GM hydrogel, but still higher than the swelling ratio of the other reported electroconductive hydrogels.^[^ ^34^ ^]^ An ideal synthetic soft tissue electroconductive hydrogel would preserve electrical functionality while matching the mechanical properties of the native nervous tissue. As previously reported, the proliferation and differentiation of NSCs can be modulated by the mechanical properties of the materials.^[^ ^7^ ^]^ It has been established that NSCs exhibit poor survival in very soft (<0.1 kPa) or very hard (>100 kPa) materials and tend to differentiate into neurons in reasonably soft materials (≈0.1–1 kPa) and into glial cells in slightly stiffer materials (≈7–10 kPa). The storage moduli of GM‐based or PPy‐based electroconductive hydrogels applied in the field of myogenic differentiation, skeletal muscle regeneration, and neural tissue engineering ranges from 10^2^–10^4^ Pa.^[^ ^35^, ^36^ ^]^ In this study, the storage moduli of the GMPE hydrogel was 1056.0 ± 133.1 Pa, which matched neural tissue mechanics (600–3000 Pa) and indicates its suitability for soft nerve tissues engineering.^[^ ^7^ ^]^ The conductivity of GMP (1.83 × 10^−3^ S cm^−1^) and GMPE (1.49 × 10^−3^ S cm^−1^) hydrogels were both superior to that of the GM hydrogel. The conductivity of the GMP and GMPE hydrogels were consistent with similar GM‐based or PPy‐based electroconductive hydrogels (10^−4^–10^−2^ S cm^−1^, Table S4, Supporting Information).^[^ ^37^, ^38^, ^39^, ^40^, ^41^ ^]^ Notably, the conductivity of our GMP and GMPE hydrogel were also close to that of normal tissue (from 5 × 10^−5^–1.6 × 10^−3^ S cm^−1^), which is beneficial for injury repair.^[^ ^42^ ^]^ Moreover, our GMPE hydrogel also showed good swelling, mechanical, and electrical stability for 14 days in physiological medium, which could facilitate their long‐term use for nerve repair. Both GMP and GMPE hydrogels had low impedance. The high conductivity ability and low resistance of GMPE and GMP hydrogels are important for nerve cell intercellular signal transmission.^[^ ^14^ ^]^ The conductivity of PPy in GMP or GMPE hydrogels can directly stimulate voltage‐gated Ca^2+^ channels to increase the intracellular Ca^2+^ levels. Intracellular Ca^2+^, acting as a second messenger, was responsible for various cellular function, such as survival, proliferation/growth, and differentiation in numerous cell types. Calcium/calmodulin‐dependent protein kinase II (CaMKII) acts as a multifunctional serine/threonine kinase which functions through Ca^2+^ signaling to negatively regulate PTEN protein, a major intrinsic impediment to axonal regeneration. After PTEN expression was inhibited, axon regeneration was promoted in corticospinal neurons by upregulation of mTOR activity.^[^ ^43^, ^44^ ^]^ Our in vitro results demonstrated that the GMP hydrogel significantly increased neuron and oligodendrocyte differentiation but reduced astrocyte differentiation in comparison with control and GM groups. Compared to the GMPE group, GMP yielded better performance in the Tuj‐1 level during the in vitro study. The higher expression of Tuj‐1 in GMP hydrogels in vitro may be accounted for its higher conductivity. Six weeks after the electroconductive hydrogels were implanted into the spinal cord hemisection gap, the GMP and GMPE hydrogels induced migration of nestin‐positive endogenous NSCs into the injury site. Both hydrogels were surrounded by neurons that even invaded into the hydrogels. Meanwhile, no significant difference in the Tuj‐1 level was observed between the two groups in vivo. In vivo, exosomes released from GMPE could inhibit the inflammation at the early stage, providing a good environment for long‐term Tuj‐1‐positive neuronal regeneration. Conversely, the SCI and GM groups formed an astrocyte scar surrounding the lesion site and no obvious neurons were observed. Lesion site NSC recruitment and neuronal regeneration subsequently promotes neuron maturation and lead to the formation of a nascent functional synaptic network.^[^ ^45^ ^]^ Oligodendrocyte regeneration and remyelination is another important process for nerve function recovery. However, spontaneous remyelination often fails after SCI, primarily due to failure of oligodendrocyte regeneration rather than oligodendrocyte depletion.^[^ ^46^, ^47^ ^]^ Hence, electroconductive hydrogels significantly improve oligodendrocyte regeneration that is critical for the myelin formation, which is essential for rapid and efficient action potential propagation and functional recovery.^[^ ^47^, ^48^ ^]^ Our results showed plenty of myelin‐associated axonal regeneration was guided by the GMP hydrogel into the lesion site. In comparison with the GM and control groups, the GMP group showed downregulation of PTEN levels and upregulation of p‐PI3K, p‐AKT, and their downstream signaling pathway, indicating that the electroconductive hydrogels modulated NSC differentiation and axonal regeneration possibly through the PTEN/PI3K/AKT/mTOR pathway.

The inflammatory responses induced by SCI limits the therapeutic efficiency of electroconductive biomaterials. In this study, we found that electroconductive hydrogels significantly aggravated the inflammation after acute SCI by increasing the proportion of M1 microglia and increased the number of CD68‐positive microglia in comparison with that in the SCI group 7 days post‐surgery. Microglia mediated‐inflammation contributes to adverse host immune effects and can lead to the rejection of transplanted biomaterials used for SCI repair.^[^ ^49^ ^]^ Recent studies suggest that the inflammation microenvironment of the injured spinal cord contributes to the transformation of anti‐inflammatory M2 microglia into proinflammatory M1 microglia shortly after injury and can last for weeks or months, which can act as a barrier to neural regeneration.^[^ ^50^, ^51^ ^]^ Therefore, decreasing early‐stage inflammation and regulating M1/M2 microglia polarization is critical for the application of engineered materials used in SCI repair. Previous studies have reported that BMSC therapies have immunomodulatory effects in severe clinical inflammatory diseases such as pancreatitis, colitis, and focal cerebral ischemia. BMSC‐exosomes are important paracrine soluble factors that act as main regulators of intercellular communication. Exosomes range in size from 10 to 100 nm and contain proteins, mRNA, and miRNA molecules that negatively regulate relative gene expression on the post‐transcriptional level.^[^ ^52^ ^]^

In this study, we combined an electroconductive hydrogel scaffold with BMSC‐exosomes for SCI therapy. The driving force for the interaction between TA and BMSC‐exosomes the reversible noncovalent hydrogen bonds formed between the phosphate group in the phospholipids and polyphenol groups in TA that allowed the exosomes in the GMPE hydrogel to be sustainably released and detected for up to 14 days in vitro. Furthermore, NSCs and BV2 cells cultured on the GMPE hydrogel could normally phagocytize exosomes released from the hydrogel, indicating that exosomes still showed good activity. Meanwhile, in vivo GMPE hydrogel implantation showed local site exosomes retention. Exosomes expressed high levels of anti‐inflammatory‐related miRNAs, including miR199a, miR99a, miR146a, miR181a, and miR411, with the miR199a level being the highest. After BV2 took in the exosomes released from the GMPE hydrogel, the levels of all miRNAs increased, especially miR‐199a, which showed a nearly threefold increase in its levels. Previous reports showed that miR‐199a acts as a negative regulator of IKK*β* and activates the IKK‐*β*‐NF‐*κ*B signaling pathway, which is the master regulator of innate immunity.^[^ ^53^, ^54^ ^]^ We found that the GMPE hydrogel promoted microglial polarization in vitro and that the expression levels of p‐IKK*α*/*β*, p‐I*κ*B*α*, and p‐P65 significantly decreased in BV2 cells grown on the GMPE hydrogel when compared with the GMP hydrogel. In addition, BAY 11–7082 was used to inhibit phosphorylation of I*κ*B*α*, which resulted in the downregulation of iNOS and upregulation of Arg‐1 in BV2 cells cultured on the GMP hydrogel. Therefore, we suggest that the GMPE hydrogel modulated microglia mediated‐inflammation, possibly through miRNAs carried by exosomes that suppressed NF‐*κ*B pathway activation. Furthermore, the in vivo results also demonstrated that GMPE hydrogel promoted fewer CD68‐positive microglia than the GMP, GM, and SCI treatments at the early stages of injury. Alarming, we also found that the GMP hydrogel promoted CD68‐positive microglia to localize to and further induce early inflammation at the injury site. The expression of iNOS was highest and Arg‐1 was lowest in the GMP group compared to the other treatment conditions, which indicated that the GMP hydrogel could promote microglia M1 polarization in vivo. These findings indicate that the combined hydrogel‐exosomes treatment largely resolved microglia mediated‐inflammation at the injury site by reducing CD68 protein levels and proinflammatory factor release. BMSC‐exosomes can minimize the deleterious effects of inflammation during the early stages, providing a good environment to ensure long‐term neuronal regeneration.

In addition, the delivery of BMSC‐exosomes in electroconductive hydrogels can exert synergistic therapeutic effects on SCI. A review of some literature shows that BMSC‐exosomes significantly enhanced axonal growth and neovascularization, while reducing microgliosis and astrogliosis, resulting in significantly functional recovery in rats with complete SCI.^[^ ^21^, ^55^, ^56^ ^]^ BMSC‐derived exosomes exhibited high expression levels of remyelination‐related miRNAs (miR145a, miR9‐5p, miR148b, miR138, miR338, miR219a, and miR1a) and axonal regeneration‐related miRNAs (miR29a, miR21, miR29c, miR26a, miR20a, miR9‐5p, miR182, miR128, miR133b, miR431, and miR124). Similar to the GMP hydrogel, the GMPE hydrogel also promoted NSC neuron and oligodendrocyte differentiation but inhibited astrocyte differentiation. Interestingly, the GMPE group showed significantly greater oligodendrocyte differentiation than the GMP group in both in vitro and in vivo studies. Two miRNAs (miR145a and miR148b) were primarily related to oligodendrocyte maturation. miR145 is a critical regulator of the human analog of myelin regulatory factor, mainly responsible for oligodendrocyte differentiation and maturation.^[^ ^24^ ^]^ The GMPE hydrogel yielded better performance in promoting NF‐positive axonal regeneration due to the addition of BMSC‐exosomes with high expression levels of axonal regeneration‐related miRNAs. MiR29a, miR21, miR29c, and miR26a also promote axon growth by suppressing PTEN and activating the PI3K/AKT pathway.^[^ ^53^ ^]^ Interestingly we found that miR29a, miR21, miR29c, and miR26a were highly expressed in BMSC‐exosomes, especially after NSC phagocytized the exosomes. Importantly, miR21 experienced a fourfold upregulation. Meanwhile, after mTOR was selectively inhibited by siRNA and Rp, the length of the axon significantly decreased in GMPE hydrogels. Therefore, BMSC‐exosomes combined electroconductive hydrogels promoted SCI repair mainly through increased NSC oligodendrocyte differentiation and myelin‐associated axonal regeneration. Furthermore, we speculated that the GMPE hydrogel promoted myelin‐associated axonal regeneration by electroconductive hydrogels and exosomes coactivating the PTEN/PI3K/AKT/mTOR pathway.

Improved myelin‐associated axonal regeneration and reduced cavitary areas may explain how neural function repaired for locomotion was recovered by GMPE hydrogel treatment. GMP can partly promote myelin‐associated axonal regeneration. Four weeks after GMP hydrogel treatment, the BMS score of the right hindlimb was significantly higher than that of the GM and SCI groups. However, it was still significantly lower than that of the GMPE hydrogel, which may be due to the synergistic effects of the embedded exosomes on axonal regeneration. Six weeks after SCI, the right hindlimb behavior in the SCI and GM groups exhibited extensive ankle movement, while mice treated with the GMP hydrogel presented only limited weight bearing. By contrast, mice in the GMPE group presented weight bearing ability and partially restored coordination 6 weeks after spinal cord hemisection. Similarly, the amplitude of CMAPs signal recorded from the gastrocnemius of the GMP group mice was significantly higher than that of the GM and SCI groups, while it was still significantly lower than that of the GMPE group. In addition, due to the early anti‐inflammatory effects of GMPE hydrogel, mice treated with GMPE hydrogel showed ankle movement just 2 week post‐SCI and their BMS scores were significantly higher than those of the SCI, GM, and GMP groups at same timepoint, further indicating that the GMPE hydrogel can promote faster and better functional recovery after SCI.

Our GMPE hydrogel also had good biocompatibility and suitable biodegradability. The conducting components in current electroconductive hydrogels, such as electroconductive polymers, metal nanoparticles, or carbon‐based materials, have very low biodegradability and poor biocompatibility.^[^ ^17^, ^43^ ^]^ A nondegradable composition would require secondary surgery to remove the material from the injury site, which may inhibit tissue regeneration at the lesion site.^[^ ^12^ ^]^ By contrast, degradable electroconductive hydrogels that match the tissue regeneration process can create a regeneration‐promoting microenvironment and guide the replacement of the biomaterials with regenerating tissue. In this study, the GM hydrogels completely degraded in 6 weeks after implantation, suggesting enzymatic cleavage of the GM backbone.^[^ ^57^ ^]^ Our dual‐network GMP hydrogel was synthesized by growing PPy chain doped with TA onto the surface of a porous GM backbone with PPy component is constructed with interconnected nanoparticles. HE staining demonstrated that monocytes around the GMP and GMPE hydrogel could phagocytose PPy and accelerate PPy chain degeneration. Hence, we believe that the degeneration of our electroconductive hydrogels was likely a result of both enzymatic hydrolysis of the GM backbone and subsequent breakdown of the PPy chain into PPy nanoparticles. Previous study demonstrated that PPy nanoparticles biosafety in vivo and that they are taken up by mouse macrophage RAW264.7 cells.^[^ ^14^, ^58^ ^]^ Our histological staining also showed that the GMPE and GMP hydrogels were progressively biodegraded at the SCI lesion site, and that neurons penetrated through the hydrogels to gradually replace them. Although the subcutaneous macroscopic view of the hydrogels implanted site showed that GMPE and GMP hydrogels were still present 6 weeks after subcutaneous implantation, the volume of these hydrogels was significantly reduced compared to that of initial implantation. Thus, nerve regeneration, which usually requires several months or years, could benefit from the slow degradability of electroconductive hydrogel implants.^[^ ^14^ ^]^ The degradation rate and mechanism of GM are obviously different from PPy under physiological conditions. Therefore, the degradation rate of the electroconductive hydrogels can be controlled by tuning their composition to match the neural regeneration process.

Additionally, degradation products of all three hydrogels showed no toxicity to the main internal organs (heart, liver, spleen, lung, and kidney) in the mouse model. Furthermore, the levels of ALT, AST, and TP in the mice treated with the three different hydrogels were similar to those in healthy mice. In vitro and in vivo data demonstrated that the long‐term biocompatibility and suitable biodegradability of exosome‐loaded electroconductive hydrogels are suitable for SCI repair. In future studies, a long‐term in vivo study will be applied to assess the complete degradation process of our hydrogel implant.

## 4 Conclusion

In summary, we have demonstrated that by immobilizing the BMSC‐exosomes in the electroconductive hydrogels prepared in our study, inflammation inhibition, NSCs recruitment enhancement, and neuronal and myelin‐associated axonal regeneration promotion can be achieved for SCI therapy. With the reversible noncovalent binding, the BMSC‐exosomes carried on the GMPE hydrogel showed good activity and could be sustainably released, allowing their accumulation in the mice spinal cord lesion site. GMP hydrogel implantation can promote NSCs recruitment, neuronal and myelinated axonal regeneration, but it inhibits scar‐forming gliosis. However, single treatments with electroconductive hydrogels even aggravated inflammation at the injury site early after SCI, which reduced the efficacy and increased the adverse effects. Due to the immunomodulatory properties of BMSC‐exosomes, the GMPE hydrogel regulated M1/M2 polarization from an M1‐ to M2‐dominant phenotype via the NF‐*κ*B pathway. Moreover, the GMPE hydrogel could reduce CD68‐positive microglia at an early stage after SCI, attenuating the adverse immune effects. In comparison with treatments using the GMP hydrogel alone, the GMPE hydrogel, a combination of exosomes and electroconductive hydrogels, further enhanced oligodendrocyte differentiation and myelin‐associated axonal regeneration. Furthermore, in vitro and in vivo studies supported our findings showing that the GMPE hydrogel enhanced neuronal and oligodendrocyte differentiation of NSCs and promoted axon growth via exosomes and electroconductive hydrogels coactivating PTEN/PI3K/AKT/mTOR pathway. Thus, the GMPE hydrogel targeted three areas, namely, reduction of early inflammation, enhancement of NSCs recruitment, and promotion of neuronal and myelin‐associated axonal regeneration, which synergistically promoted locomotor recovery after mice spinal cord hemisection. This study suggests that a combination of electroconductive hydrogels and BMSC‐exosomes would be a promising therapeutic strategy for SCI treatment.

## 5 Experimental Section

### Isolation and Characterization of Mouse Bone Marrow Mesenchymal Stem Cells (BMSCs)

Bilateral humeri and femurs of C57BL/6J mice (*n* = 5, 4‐week‐old) were extracted under sterile conditions to resect metaphysics of each bone. Low glucose Dulbecco's modified Eagle medium (DMEM, Gibco) containing 10% FBS and 1× penicillin/streptomycin was used to rinse the bones until the color of bone marrow tuned white. The mixture was then incubated at 37 °C with 5% CO_2_. The culture medium was renewed every three days to remove nonadherent cells. Surface marker antibodies were used as described previously to verify cells expressing CD11b, CD45 CD29, CD90, and CD44 (eBioscience). The differentiation of adipocytes, osteoblasts, and chondrocytes of extracted BMSCs was included as described previously.^[^ ^59^ ^]^ Briefly, BMSCs were cultured in 1) osteogenic differentiation medium: DMEM containing 10% FBS, 50 µg mL^−1^ ascorbic acid, 10 nmol L^−1^ dexamethasone, and 10 mmol L^−1^ *β*‐glycerophosphate; 2) adipogenic differentiation medium: DMEM containing 10% FBS, 50 µg mL^−1^ ascorbic acid, 10 µg mL^−1^ insulin, 10 nmol L^−1^ dexamethasone, and 50 µg mL^−1^ indomethacin; or 3) chondrogenic differentiation medium: DMEM containing 1% FBS, 50 µg mL^−1^ ascorbic acid, 100 nmol L^−1^ dexamethasone, 10 ng mL^−1^ transforming growth factor alpha (TGF‐*β*), 6.25 µg mL^−1^ insulin, 6.25 µg mL^−1^ transferrin, 6.25 µg mL^−1^ selenous acid. The media were changed every 3 days. Osteoblasts, adipocytes, and chondrocytes were identified using Alizarin Red staining, Oil Red O staining, and Alcian Blue staining, respectively, 14 days after culture.

### Isolation and Characterization of BMSC‐Exosomes

All BMSC‐exosomes used in the in vitro and in vivo experiments were obtained from the same batch of BMSCs. FBS was ultracentrifuged at 120 000 × *g* under 4 °C for 12 h using an ultracentrifuge (Beckman Coulter) to prepare exosome‐free FBS. Exosome‐free culture medium was used to replace conventional culture medium as the cell density reached 80%. After the culture medium was gradient centrifuged to remove dead cells and cell debris, the supernatant was centrifuged at 100 000 × *g* for 90 min, and the isolated exosomes were resuspended in 50 µL PBS (Gibco) for further use. The NTA assay was used to detect the size of exosomes, and TEM (HT7700, HITACHI) was used to analyze the morphology of exosomes. WB was used to determine the expression of Flotillin‐1 (Abcam), TSG101 (Abcam), and CD63 (ProteinTech). A BCA protein assay kit (Thermo Fisher) was used to detect the concentration of exosomes.

### Data Retrieval, Extraction, and Analysis of GEO Datasets

miRNA expression in exosomes secreted by mouse bone marrow stem cell (MSC) was retrieved from the GEO database using keywords “microRNA,” “exosomes,” “MSC,” and “murine.” Dataset GSE181530 (Baral et al., 2021, PMID: 34479773) was analyzed via the GPL19057 platform (Illumina NextSeq 500 (Mus musculus)). GSE164965 (Jin et al., 2021, PMID: 34090522) was analyzed via GPL17021 platform (Illumina HiSeq 2500 (Mus musculus)). GSE119790 (Zhang et al., 2019, 40, PMID: 30866952) was analyzed via GPL22383 platform (Agilent‐070155 Mouse miRNA Microarray). miRNAs in untreated MSCs‐derived exosomes in each dataset were selected for further analysis. The expression of miRNAs was analyzed using the R package heatmap.

### MicroRNA Gene Expression

The miRNeasy Mini Kit (Qiagen) was used to extract miRNAs from exosomes. The primer information for each miRNA is provided in Table S2 of the Supporting Information. U6 was set as the normalized miRNA expression. *E. coli* Poly(A) Polymerase (NEB) was used to catalyze the template‐independent addition of AMP from ATP to the 3′ end of miRNA. Reverse transcription for cDNA synthesis was performed using the PrimeScript TM RT reagent Kit (Takara). Reverse transcription quantitative polymerase chain reaction (RT‐qPCR) was performed using LightCycler 480 SYBR Green I Master (Roche).

### Synthesis of GM, GMP, and GMPE Hydrogel

GM monomer was synthesized as previously described.^[^ ^7^ ^]^ Briefly, 1 g gelatin was dissolved in 10 mL PBS (pH 7.4) at 50 °C to obtain 10% (w/v) gelatin. Then, 0.5 mL methylacrylic anhydride was added into the gelatin solution and incubated for 1 h with stirring. Another 10 mL PBS was introduced to stop the reaction. The solution was dialyzed using deionized water and lyophilized for future use. The GM hydrogels were prepared by UV cross‐linking of the GM monomer (3% w/v) and 0.5% photoinitiator (Irgacure 2959; Sigma). Solution I was prepared by dissolving 0.03 g TA and 70 µL pyrrole (Py) in 10 mL deionized water. Solution II was prepared by dissolving 0.228 g APS (Sigma) in 10 mL deionized water. The two solutions were then stored at 4 °C for 30 min before use. The GM hydrogels were immersed in solution I for 12 h and then solution II was added and the mixtures was incubated at 4 °C overnight to synthesize the GMP hydrogel. GMP hydrogels were then incubated in DMEM basic overnight at 4 °C to clear any un‐cross‐linked side products. Finally, 200 µg of BMSC‐exosomes was dropped onto 50 µL hydrogel and incubated at 4 °C overnight to obtain the GMPE hydrogel.

### Characterization of Hydrogel Physical Properties—SEM Analysis

After the hydrogels were frozen at −20 °C for 24 h, they were transferred to a freeze drier under vacuum at −80 °C for 48 h. Hydrogel samples were flash‐frozen by using liquid nitrogen to preserve their original internal cross‐linked structure before the samples were sputter coated with platinum (Pt) for 60 s. SEM (Quanta 200, FEI) was used for morphological observation at 10 kV accelerating voltage.

### Characterization of Hydrogel Physical Properties—^1^H NMR of GM Hydrogel

Proton nuclear magnetic resonance (^1^H NMR) spectroscopy was used to evaluate the chemical modification of gelatin. Gelatin and GM were dissolved in D_2_O at a concentration of 30 mg mL^−1^. A Bruker Avance 400 spectrometer was used to record ^1^H NMR spectra from 16 scans at a ^1^H resonance frequency of 400 MHz.

Characterization of Hydrogel Physical Properties—*Fourier Transform Infrared Spectroscopy (FTIR)*: Spectra of GM, GMP, and GMPE hydrogels were detected using an Avatar 380 FTIR spectrometer (Thermo Nicolet). After the samples were freeze‐dried at −80 °C for 24 h, each hydrogel was ground into a powder by grinding after liquid nitrogen processed. Samples were pressed into pellets and recorded by the spectrum software.

### Characterization of Hydrogel Physical Properties—Rheological Experiments

Rheological properties were measured using a rotary rheometer (Physician MCR301, Anton Paar). Angular frequency sweep (0.1–100%) was conducted at a fixed strain of 1%. After 1 mL of each hydrogel was placed onto the lower plate of the machine and preheated to 37 ℃, the upper plate was subsequently lowered to a gap of 1 mm to begin the rheological properties measurement.

### Characterization of Hydrogel Physical Properties—Peeling Adhesion Test

A 180° peeling test was employed to evaluate the adhesive strength of GMPE. GMPE (10 × 1 × 120 mm^3^) was adhered to a porcine skin with one end open. Krazy Glue was employed to bind the back of GMPE with a polyethylene terephthalate (PET) to prevent GMPE from breaking during the test. A dynamic mechanical analyzer (DMA Q800, USA) was employed to perform the peeling test with a rate of 100 mm min^−1^.

### Characterization of Hydrogel Physical Properties—Swelling Property

Each hydrogel was immersed in PBS to achieve full swelling at room temperature before the wet weight (W1) was recorded. The surface water of each hydrogel was absorbed by filter paper before the hydrogels were freeze‐dried under vacuum at −80 °C for 48 h, and the dried weight (W2) was recorded. The swelling property of each hydrogel was measured using following formula

(1) $$\mathrm{Swelling}\;\mathrm{ratio}\left(\%\right)=\frac{W1-W2}{W2}\times 100$$

### Characterization of Hydrogel Physical Properties—Electrical Characterization

Electrical characterization including CV, EIS, and *I*–*V* of each hydrogel was performed using an electrochemical workstation (Zennium Zahner).^[^ ^14^ ^]^ The working electrode was a hydrogel‐coated ITO glass. The counter and reference electrodes were a platinum mesh and Ag/AgCl, respectively. The CV measurements were performed at a scan rate of 10 mV s^−1^, ranging from −0.5 to 1.0 V in 0.1 m PBS. The EIS spectra were measured at open circuit potentials ranging from 100 kHz to 0.01 Hz. The *I*–*V* was tested using a two‐probe Keithley 2400 Sourcemeter in the range from −0.5 to 0.5 V.

### In Vitro Studies—NSC Isolation and BV2 Cell Culture

Brains from E14 mouse embryos were dissociated into single cells with mechanical shearing and transferred into low‐attachment dishes. Cells were cultured in DMEM/F12 (Gibco) supplemented medium containing with 1 × B27 neuronal supplement (Gibco), 20 ng mL^−1^ epidermal growth factor (EGF; PeproTech), 20 ng mL^−1^ basic fibroblast growth factor (bFGF; PeproTech), 1× Glutamax (Gibco), and 1× penicillin/streptomycin (Gibco). The medium was changed every 3 days. The BV2 cells were purchased from the ATCC cell bank and cultured in high glucose DMEM medium containing 10% FBS, which was replaced every other day.

### In Vitro Studies—DRG Obtained and Cultured

DRG neurons were extracted from 5 day old postnatal mouse. Briefly, the pups were decapitated and the spinal canal was dissected horizontally. After DRGs were fetched under a stereomicroscope, they were cultured on hydrogels in a serum‐free culture medium containing neurobasal medium (Gibco), 50 ng mL^−1^ NGF, 1 × B27 neuronal supplement (Gibco), 1× penicillin/streptomycin (Gibco), and 1× Glutamax (Gibco) with 5% CO2 at 37 °C. The culture medium was changed every other day. The axonal lengths and densities were measured using the Simple Neurite Tracer plugin from ImageJ software which can trace and register individual branches with respect to the cell body.^[^ ^60^ ^]^

### In Vitro Studies—Exosomes Labeling and Cell Phagocytosis Studies

The PKH26 red fluorescent membrane linker dye (Sigma) was used to label BMSC‐exosomes. To avoid the PKH26 aggregates, the process of exosomes staining was strictly controlled by avoiding the application of salt solutions. Exosomes were resuspended with 500 µL Diluent C solution, and then 5 µL PKH26 red fluorescent dye was added to the suspension and incubated at 37 °C for 5 min. To neutralize the residual dye, 10 mL complete DMEM medium was added to the samples. Samples were ultracentrifuged at 100 000 × *g* for 1 h at 4 ℃. The exosome precipitation was wash twice with 10 mL complete DMEM medium to eliminate the unbound dye. Also, the centrifuge tube was replaced after each centrifugation. To further detect exosomes phagocytosis, cytoskeleton staining was performed using Actin‐Tracker Green (Beyotime). Cells were fixed with 4% paraformaldehyde for 30 min, and then incubated in a membrane breaking solution containing 0.2% Triton‐100X (Biofroxx) and 6% bovine serum albumin (BSA, Biofroxx) at 37 ℃ for 1 h. Actin‐Tracker Green was added, and then samples were incubated at 4 °C for another 1 h. Hoechst 33342 (Sigma) stain was added for 5 min before samples were observed using a confocal reflection microscope (Leica).

### In Vitro Studies—NSC Viability, Proliferation, and Adhesion on the Hydrogels

The viability of NSCs was calculated using Calcein‐AM/ Propidium iodide (Calcein‐AM/PI, Invitrogen) staining 24 h after coculture with each hydrogel as previously described.^[^ ^7^ ^]^ Calcein‐AM and PI were added to double‐stain NSCs for 15 min at 37 °C in 5% CO_2_. The images of living/dead cells were captured using a confocal reflection microscope (Leica). In addition, the CCK‐8 (Dojindo) solution was added to 12‐well NSCs culture plates at a ratio of 1:10 to detect cell proliferation on days 1, 3, and 7. After incubation for 4 h, 100 µL of supernatant mixed solution was transferred into 96‐well plates and measured with an enzyme‐labeling instrument (SpectraMax M5) at the 450 nm wavelength. The cytoskeleton was stained with Actin‐Tracker Green after 3 days of culture to observe cell adhesion and spreading.

### In Vitro Studies—Gene Expression

A total RNA kit (Omega) was used to harvest mRNA from cells cultured on each hydrogel. Similarly, cDNA reverse transcription was performed using the PrimeScript TM RT reagent kit. RT‐qPCR was performed using LightCycler 480 SYBR Green I master mix. The primers used in these experiments are listed in Table S2 of the Supporting Information.

### In Vitro Studies—IF Analysis

Cells or tissues were fixed in 4% paraformaldehyde for 30 min. Subsequently, samples were incubated in PBS containing 0.2% Triton‐100X and 6% BSA for 1 h at 37 ℃. The corresponding primary antibodies were then incubated overnight at 4 °C. Secondary corresponding antibodies were added to the cells or tissues and incubated at 37 ℃ for 1 h. Hoechst 33342 was used to stain nuclei before imaging. PBS was used to wash the samples three times between each step. Images were captured using a confocal reflection microscope (Leica).

### In Vitro Studies—WB Assay

Cells or tissues were lysed in RIPA lysis buffer (CWBIO) containing protease inhibitor (Thermo Fisher) and phosphatase inhibitor (Thermo Fisher). Protein concentration was determined using a BCA protein assay kit (Thermo Fisher). Equal amounts (40 µg) of each protein suspension were separated on an 8% SDS‐PAGE gel (Thermo Fisher) and then transferred onto polyvinylidene fluoride (MILLIPORE) membranes. Membranes were subsequently blocked with 5% skim milk for 1 h before being incubated with primary antibodies at 4 °C overnight. A specific secondary antibody (CST) was added to the membranes for 1 h before the immunoblots were visualized using an enhanced chemiluminescence kit (Thermo Fisher). Total protein analysis was performed using ImageJ software.

### In Vitro Studies—RNA Interference

The small interfering RNAs (siRNAs), which targets mTOR, were purchased from RIBOBIO (China). The sequences of siRNAs are listed in Table S3 of the Supporting Information. The siRNAs are transfected into cells using Lipofectamine 3000 Reagent (Invitrogen) according to the manufacturer's instructions.

### In Vivo Studies—Ethics Statement

All experimental protocols and animal experiments were approved by the Animal Care and Use Committee of Sun Yat‐sen University and conducted in accordance with the National Institutes of Health Guide for the Care and Use of Laboratory Animals.

### In Vivo Studies—In Vivo Bioluminescence Imaging

In vivo bioluminescence imaging was used to evaluate the retention of exosomes in the mouse model. Mice were euthanized with isoflurane before they were imaged using the noninvasive In‐Vivo FX Pro (Bruke) imaging system.

### In Vivo Studies—In Vivo Degradation and Biocompatibility of the Hydrogels

Adult male C57BL/6J mice (6–8 weeks old, *n* = 27) were used for the evaluation of in vivo biodegradability and biocompatibility of each hydrogel. The mice were divided into GM hydrogel (*n* = 9), GMP hydrogel (*n* = 9), and GMPE hydrogel (*n* = 9) groups. Each hydrogel was subcutaneously inserted into the backs of mice, which were sacrificed at weeks 1, 3, and 6 after implantation. The surrounding tissue was removed and fixed in 4% paraformaldehyde for further pathological analysis with HE. The thickness of the fibrotic capsule was determined using ImageJ software.

### In Vivo Studies—Animals Spinal Cord Hemisection Model

Seventy‐five adult male C57BL/6J mice (6–8 weeks old) were randomly assigned to sham (*n* = 15), SCI (*n* = 15), GM (*n* = 15), GMP (*n* = 15), and GMPE (*n* = 15) groups. Animals were anesthetized by intraperitoneal injection of a mixture of 5 mg kg^−1^ xylazine and 70 mg kg^−1^ ketamine. A 2 mm long longitudinal right‐side spinal cord hemisection was performed at the T9‐10 level under a microscope. Then, hydrogels were transplanted into the lesion sites, which were then surgically closed. Mice bladder were emptied manually twice daily until spontaneous voiding resumed.

### In Vivo Studies—Functional Recovery and Footprint Analysis in SCI Model Mice

Locomotion recovery in the mice was evaluated using the BMS scores and footprint analysis 6 weeks after SCI. In order to assure unbiased evaluation of behavioral recovery, the behavioral recovery of mice was assessed by an independent experimenter. BMS scores were performed to assess mice hind limb behavior before the operation and then weekly after SCI. Mice were allowed to walk freely on a grid and the scores were judged based on hind limb movement function ranging from 0 (no ankle movement) to 9 (complete function recovery). Footprint analysis was applied to assess body weight support and physical coordination. The fore and hind limbs of the mice were dipped into blue and red ink, respectively. Then, the mice were allowed to walk on a paper‐covered narrow surface. The distance between the two sides of the hind paws was identified as the base of support. Stride length was characterized as the perpendicular distance between the fore and hind limbs to assess the coordination ability. The angle which formed by two lines crossing the center of the hind paw to the third toe, and the stride line was defined as the angle of rotation. The frequency of toe dragging was characterized as the ratio of dragging to total footsteps.

### In Vivo Studies—Spinal Evoked CMAPs

The CMAPs data were recorded with a BIOPAC MP160 system (BIOPAC) from mice 6 weeks after SCI. Mice were anesthetized by intraperitoneal injection of a 5 mg kg^−1^ xylazine and 70 mg kg^−1^ ketamine mixture before their limbs were fixed on a board with cloth bands. Two stimulating electrodes were inserted into the spinal cord above the injury site, and CMAPs were used to evaluate motion pathways from recording electrodes inserted into the ipsilateral gastrocnemius. A ground electrode was inserted into the mouse tail. The stimulation strength was 0.5 V and the stimulus duration was 500 ms.

### In Vivo Studies—In Vivo MRI

Mice were anesthetized by suction anesthesia and mounted in the supine position within a 7.0‐Tesla MR scanner (PharmaScan70/16 US, Bruker Biospin MRI GmbH) configured with a dedicated animal coil. Conventional MRI scans were performed at 3 and 6 weeks after surgery. For conventional MRI, a sagittal T2‐weighted image (T2WI) and a coronal T2‐weighted image (T2WI) were obtained. A spin echo (SE) sequence with the following parameters was used to acquire anatomical images: sagittal T2WIs (time of repetition (TR)/time of echo (TE) 1263 ms/25 ms, 512 × 512 matrix, field of view (FOV) 16) and coronal T2WIs (TR/TE 1263 ms/25 ms, 512 × 512 matrix, FOV 16). The volume of the lesion size was quantitatively analyzed through conventional MRI using the following formula

(2) $$\begin{array}{ccc} & & \mathrm{Volume}\\ & & =\frac{\text{slice}\;\text{number}\;\text{of}\;\text{lesion}\;\text{site}\times \text{largest}\;\text{lesion}\;\text{area}\times \text{slice}\;\text{thickness}}{2} \end{array}$$

### In Vivo Studies—Hemocompatibility of the Hydrogels

In vitro hemolysis analysis was used to evaluate the hemocompatibility of the hydrogels. Blood samples were collected in anticoagulation tubes and cocultured with each hydrogel in 37 °C water for 4 h. PBS and Triton‐100X were set as negative and positive group, respectively. After centrifugation at 10 000 × *g* for 5 min at 4 °C, the supernatant was transferred to 96‐well plates and measured using an enzyme‐labeling instrument (SpectraMax M5, 540 nm). The hemolysis percentage was calculated using the following equation

(3) $$\begin{array}{c} \mathrm{Hemolysis}\left(\%)\right)=\frac{\mathrm{sample}\mathrm{absorbance}-\mathrm{negative}\mathrm{control}}{\mathrm{positive}\mathrm{absorbance}-\mathrm{negative}\mathrm{control}}\times 100 \end{array}$$

### In Vivo Studies—Histological Analysis

Mice were deep euthanized using 0.6% sodium pentobarbital (10 g/0.1 mL, Merck) 6 weeks post‐SCI. The animals were then intracardially perfused with PBS followed by 4% paraformaldehyde. The spinal cord containing the injury site was dissected, fixed in 4% paraformaldehyde for 24 h, and embedded in paraffin. Samples were then cut into 20 µm thick sections using a Leica RM2245 electric slicer. A general review and lesion cavity assessment was performed with HE staining in samples from each spinal cord treatment group. LFB staining was used to identify remyelination at the lesion sites.

### Statistical Analysis

All values are represented as the mean ± standard deviation (SD). Statistical analysis was performed using Statistical Product and Service Solutions software (version 22.0). Repeated‐measures one‐way analysis of variance (ANOVA) followed by Bonferroni's multiple comparison test was used to compare differences between treatment groups. Differences were considered statistically significant at *p* < 0.05.

## Conflict of Interest

The authors declare no conflict of interest.

## Author Contributions

L.F., C.L., and X.C. contributed equally to this work. L.F. designed and synthesized the GMPE hydrogel under the guidance of L.Z. and C.N., L.F., C.L., and X.C. performed the in vitro and in vivo experiments. L.F. analyzed the data and wrote the manuscript with guidance from C.N., L.Z., Y.S., and L.Z.. P.Y., D.C., and G.T. provided experimental guidance. Y.Z. and H.W. helped optimize device applications, especially the mouse MRI. L.Z., Y.L., C.D., P.G. and F.L. conducted the experimental verification. C.N., L.Z., and Y.S. provided guidance during all stages of the project.

## Supporting information

Supporting Information

## Data Availability

The data that support the findings of this study are available in the supplementary material of this article.
