# Supplementary material for: Exosomes‐Loaded Electroconductive Hydrogel Synergistically Promotes Tissue Repair after Spinal Cord Injury via Immunoregulation and Enhancement of Myelinated Axon Growth
Source: Adv Sci (Weinh). 2022 Mar 6;9(13):2105586. doi: 10.1002/advs.202105586 (PMC9069372; doi:10.1002/advs.202105586)
Supplement: Supplementary file 1 — Supporting Information [file ADVS-9-2105586-s001.pdf]

ADVANCED SCIENCE
Open Access

Supporting Information

for *Adv. Sci.*, DOI 10.1002/advs.202105586

Exosomes-Loaded Electroconductive Hydrogel Synergistically Promotes Tissue Repair after Spinal Cord Injury via Immunoregulation and Enhancement of Myelinated Axon Growth

Lei Fan, Can Liu, Xiuxing Chen, Lei Zheng, Yan Zou, Huiquan Wen, Pengfei Guan, Fang Lu, Yian Luo, Guoxin Tan, Peng Yu, Dafu Chen, Chunlin Deng, Yongjian Sun, Lei Zhou* and Chengyun Ning**

Supporting Information

Exosomes-loaded electroconductive hydrogel synergistically promotes tissue repair after spinal cord injury via immunoregulation and enhancement of myelinated axon growth

Lei Fan†, Can Liu†, Xiuxing Chen†, Lei Zheng, Yan Zou, Huiquan Wen, Pengfei Guan, Fang Lu, Yian Luo, Guoxin Tan, Peng Yu, Dafu Chen, Chunlin Deng, Yongjian Sun, Lei Zhou*, Chengyun Ning**

*Corresponding author. Email: imcyning@scut.edu.cn or ning_lab@hotmail.com (C.N.);

zhoul@gzhmu.edu.cn (L.Z.); nysysyj@163.com (Y.S.)

This PDF file includes:

Figure S1 Identification of BMSCs and BMSC-exosomes.

Figure S2 Physical properties of different hydrogels.

Figure S3 In vitro and in vivo exosomes release from loaded hydrogels.

Figure S4 Data retrieval, extraction and analysis of transcriptomic miRNAs expression in BMSC-derived exosomes from the Gene Expression Omnibus (GEO) database.

Figure S5 In vitro biocompatibility of hydrogels.

Figure S6 In vivo biodegradability of implanted hydrogels.

Figure S7 In vivo biocompatibility of implanted hydrogels.

Figure S8 Microglia M1/M2 polarization was evaluated after p-IκBα was inhibited with BAY 11-7082 treatment.

Figure S9 NSC axon outgrowth on GMPE hydrogels after selective p-mTOR inhibition.

Figure S10 Spinal cord pathology after SCI at 3 and 6 weeks.

Table S1. Serum and cellular contaminants were negative in the BMSC-derived exosomes detected by the proteomic mass spectrometry.

Table S2. Primer sequences of each gene was shown below.
Table S3. Sequences of three siRNAs were shown below.

Table S4. The comparison of conductivity.

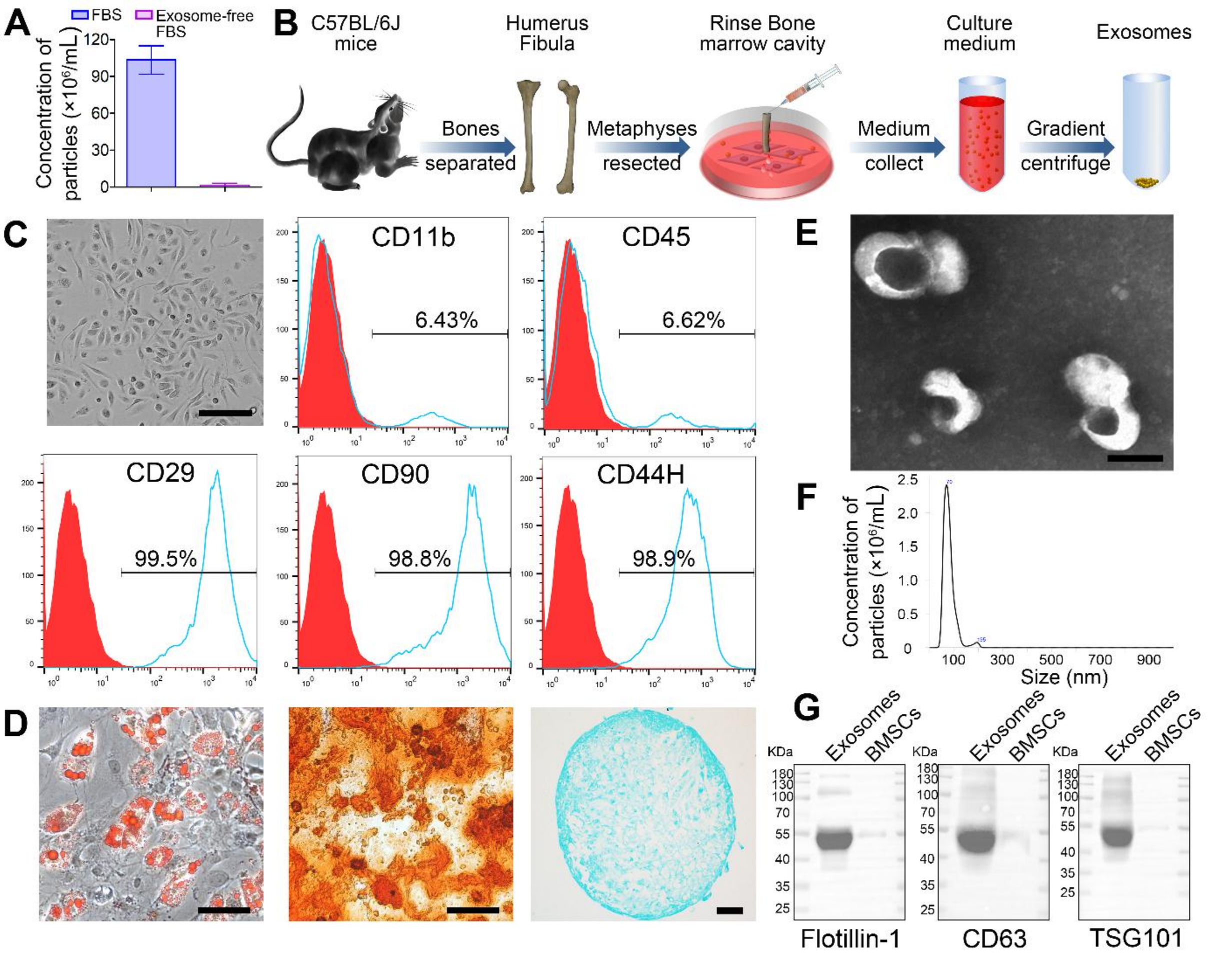

Figure S1 Identification of BMSCs and BMSC-exosomes. (**A**) NTA assay was used to determine the concentration of exosomes and microvesicles in the FBS before and after ultracentrifugation. (**B**) Illustration of the mouse BMSCs and exosomes extraction process. (**C**) Photomicrographs illustrating the spindle-like morphology of primary BMSCs Scale bars, 100 µm. Cells expressed the MSC-positive surface markers such as CD29, CD90, and CD44H, but not the negative surface markers such as CD11b and CD45. (**D**) Cells cultures differentiated into osteocytes, adipocytes, and chondrogenesis. Scale bars, 50 µm. (**E**) Transmission electron microscopy showing exosomes with a cup-shaped morphology. Scale bars, 100 nm. (**F**) NTA analysis showing exosomes with a particle size of 70 to 140 nm. (**G**) Full-sized images of WB results showing that exosomes positively expressed CD63, TSG101, and Flotillin-1 proteins.

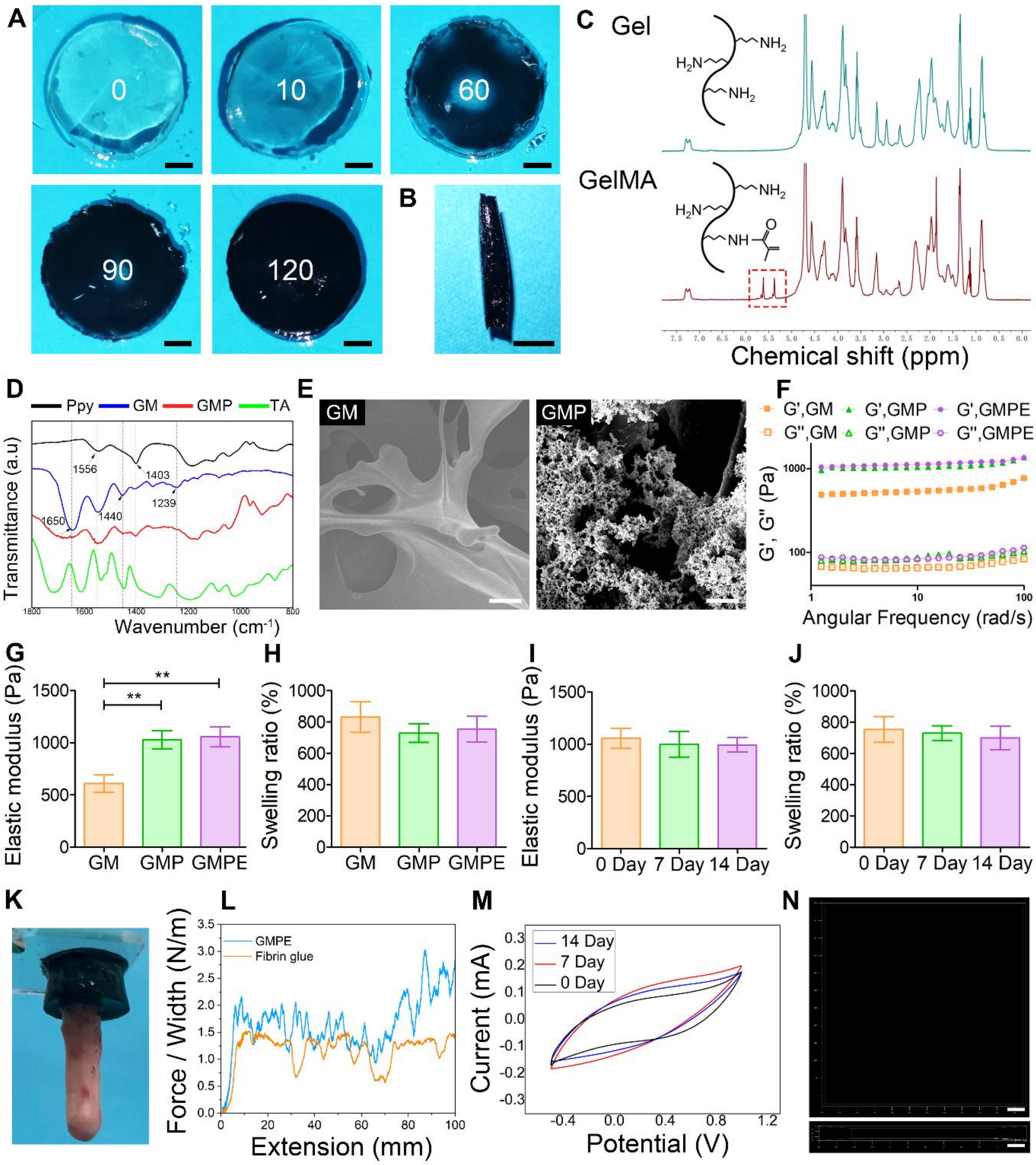

Figure S2 Physical properties of different hydrogels. (**A**) The transparent GM hydrogel gradually became black over time (in minutes) with the formation of PPy polymers. Scale bar: 5 mm. (**B**) Cross sectional images of the GMP hydrogel incubated for 2 h were black. (**C**) ^{1}H NMR spectrum characterization of GM hydrogels showed two new peaks at 5.3 ppm and 5.5 ppm when compared to gelatin. (**D**) GMP hydrogels exhibited the characteristic gelatin amide bands, including C=O stretching at 1650 cm^{-1} (amide I), C–N at 1440 cm^{-1}, and N-H deformation at 1239 cm^{-1} (amide III), as well as the Py ring vibrations peaks at 1556 cm^{-1} and 1403 cm^{-1}. (**E**) A high magnification SEM of GM, GMP, and GMPE hydrogels showing the interconnected globular nanoparticle morphology of PPy that aggregates onto the GM backbone. Scale bars, 10 µm. (**F**) The mechanistic properties of all samples were tested by dynamic oscillatory frequency sweep measurement that showed storage moduli (elastic modulus, G′) of all hydrogels were larger than the loss moduli (viscous modulus, G″) over an angular frequency range of 1-100 Hz, indicating that the hydrogels had good stability. (**G**) Graph of the quantification of GM, GMP, and GMPE hydrogels mechanical properties (n=5).

(**H**) Equilibrium swelling properties of GM, GMP and GMPE hydrogels (n=5). (**I**) GMPE hydrogels exhibited mechanical stability 14 days after soaking in physiological medium (n=5). (**J**) GMPE hydrogels also exhibited swelling ratio stability 14 days after soaking in physiological medium (n=5) (**K**) Mouse spinal cord tissue could stick to the GMPE hydrogels in vitro. (**L**) Force/width-extension curves of the peeling adhesion test. (**M**) The CV curve of GMPE hydrogel showed its stable electrochemistry 14 days after soaking in physiological medium. (**N**) 3D IF imaging of hydrogel loaded without exosomes labeled with PKH26 showed that almost no fluorescent PKH26 dye was residual after ultracentrifugation. Statistical differences were determined using an ANOVA with Bonferroni's multiple comparison test (* $p<0.05$, ** $p<0.01$, *** $p<0.001$).

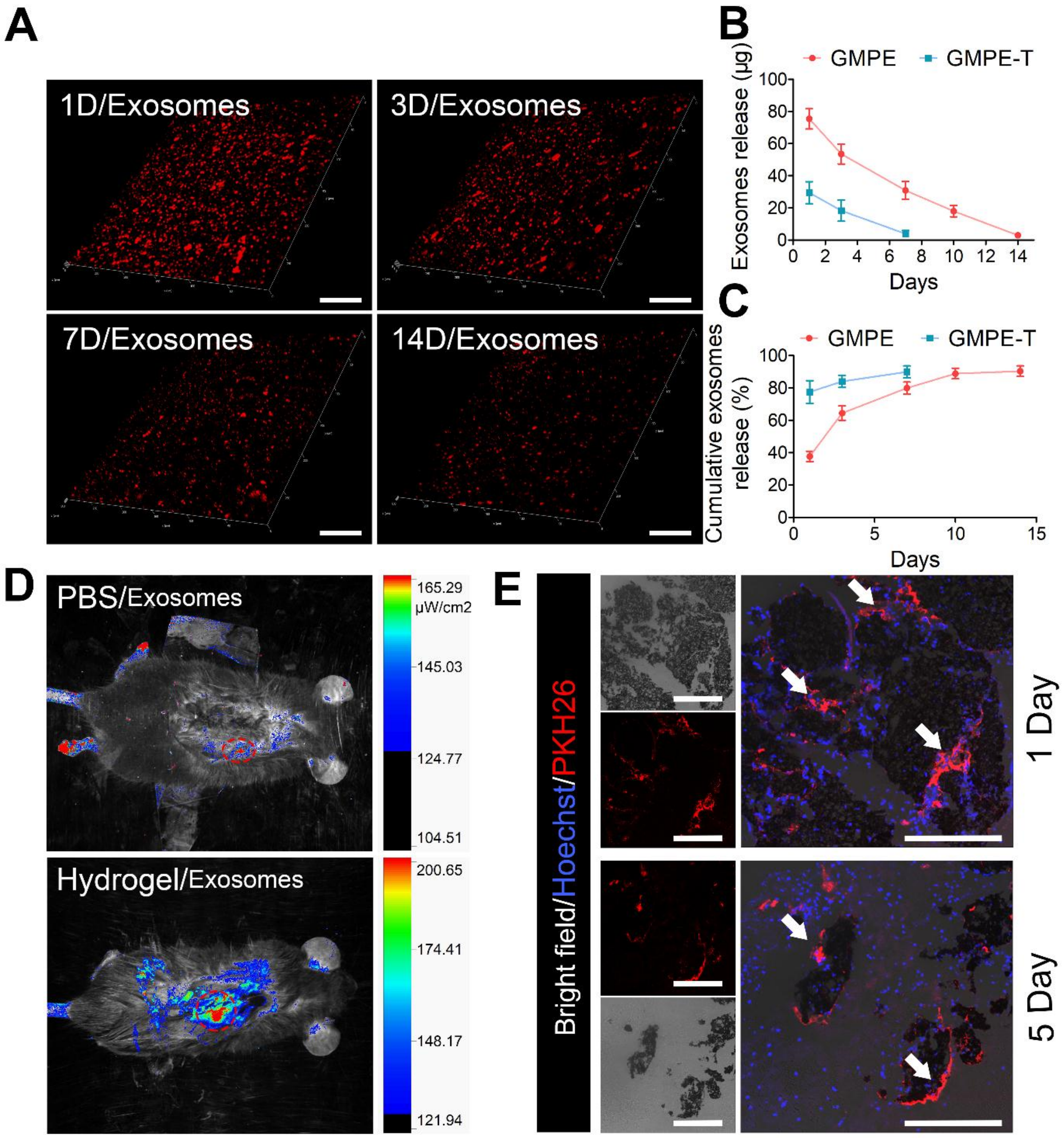

Figure S3 In vitro and in vivo exosomes release from loaded hydrogels. (**A**) IF image showing that exosomes can be detected 14 days after they were immobilized in the hydrogel. Scale bars, 50 µm. (**B**) Exosomes were continuously released into the medium supernatant from the GMPE hydrogel after 14 days (n=5). (**C**) More than 90% of the exosomes were released from the hydrogels over time (n=5). (**D**) In vivo bioluminescence imaging showed that when exosomes placed into a mouse model with hydrogel, the hydrogel significantly improves the exosomes retention at the injury site 5 days after implantation. Heatmap scale indicates $\mu W/cm^2$. (**E**) Labeled exosomes can be detected 5 days after implantation at lesion sites and endogenous cells can phagocytose exosomes released from the GMPE hydrogel. White arrows indicate exosomes taken up by the endogenous cells. Scale bars, 500 µm

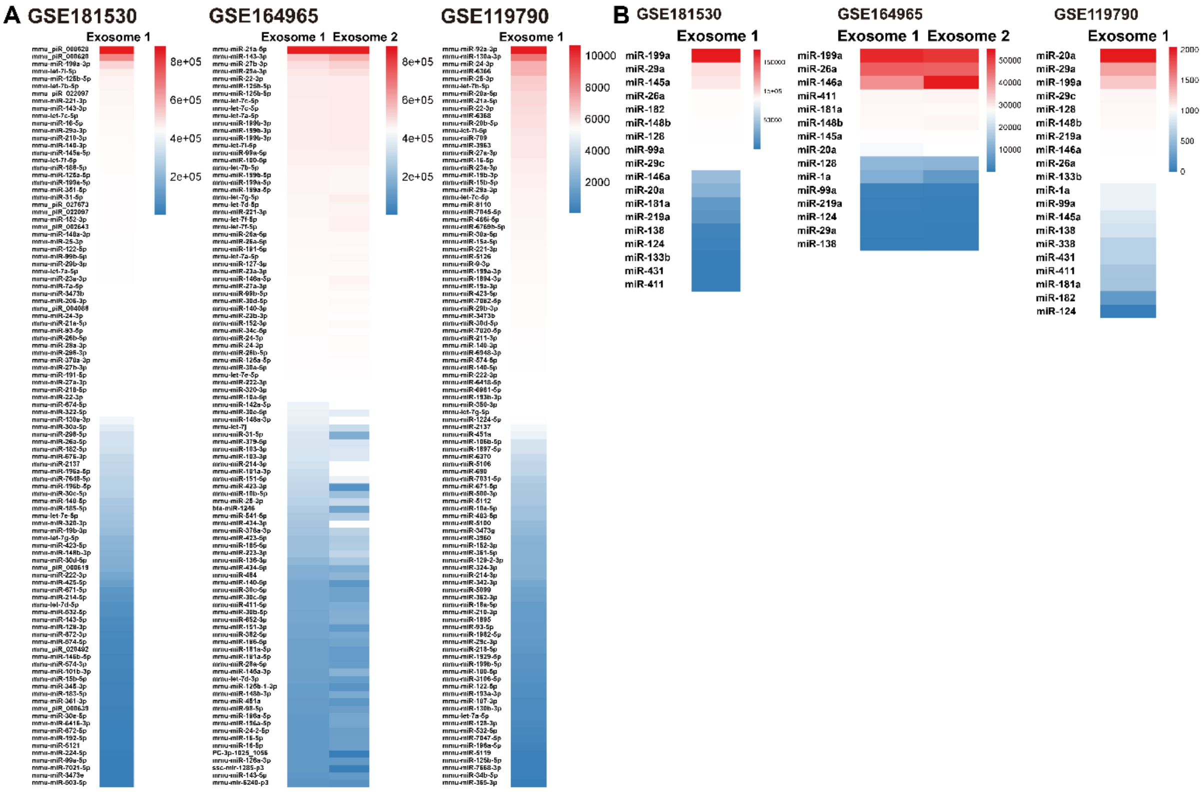

Figure S4 Data retrieval, extraction and analysis of transcriptomic miRNAs expression in BMSC-derived exosomes from the Gene Expression Omnibus (GEO) database. (**A**) Heatmap of top 100 miRNAs of three different series GSE181530, GSE164965, and GSE119790. (**B**) heatmap of selective miRNAs of three different series GSE181530, GSE164965 and GSE119790.

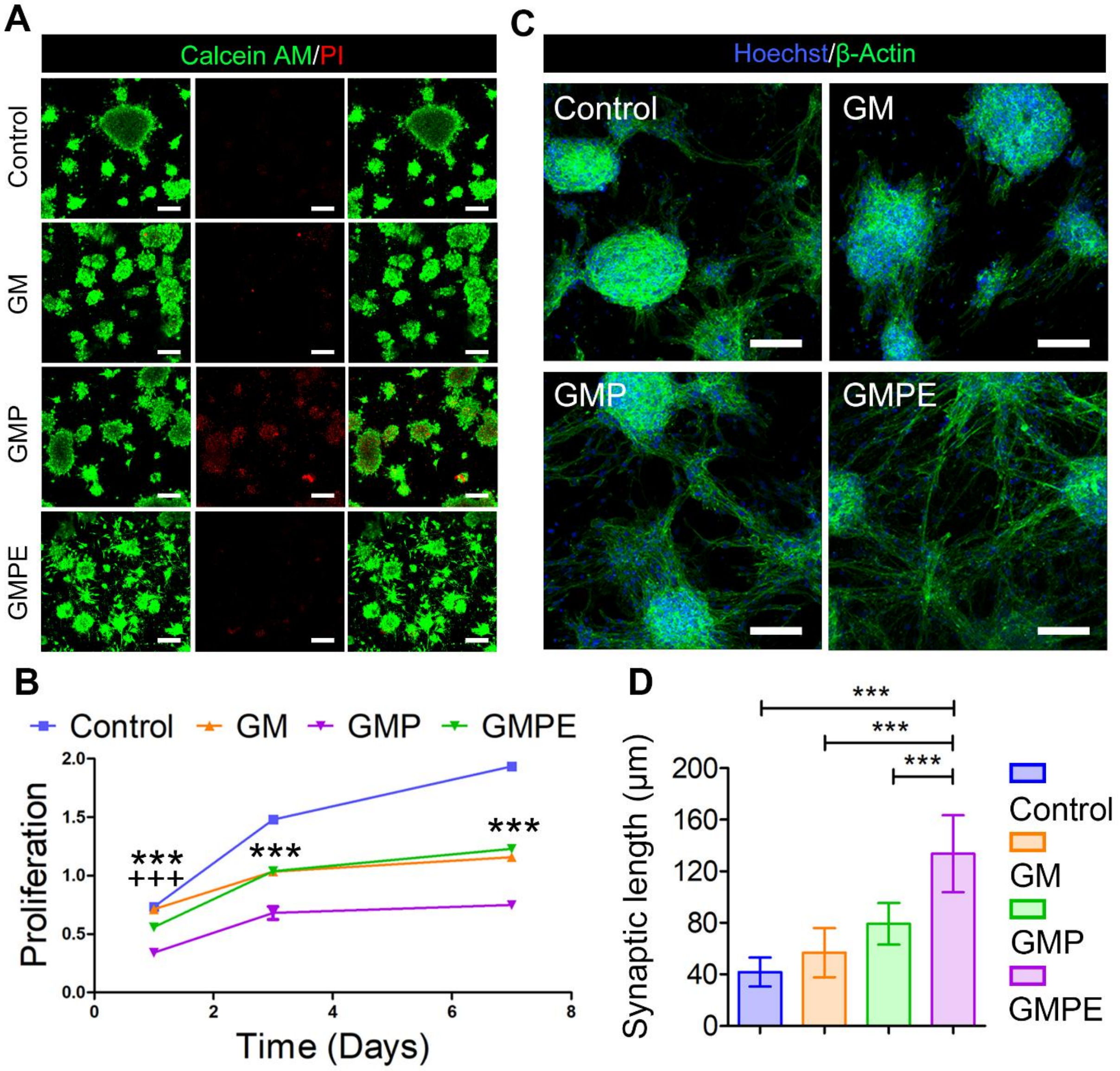

Figure S5 In vitro biocompatibility of hydrogels. (**A**) A live/dead assay in NSCs was used to evaluate the in vitro biocompatibility of hydrogels. Scale bars, 100 μm. (**B**) CCK-8 analysis of NSCs was also used to evaluate the in vitro biocompatibility of hydrogels (n=3). The proliferation of cells cultured on the GMPE hydrogel was similar to that on GM hydrogel, and the proliferation rate on GMPE hydrogel was significantly higher than that on GMP hydrogel (n=3). (**C**) Cytoskeleton staining in NSCs was also used to evaluate hydrogel adhesion. Scale bars, 100 μm. (**D**) The length of synaptic length was quantified using Image J software (n=11). Statistical differences were determined using an ANOVA with Bonferroni's multiple comparison test (GMPE compared to GMP * $p<0.05$, ** $p<0.01$, *** $p<0.001$; GMPE compared to GM, + $p<0.05$, ++ $p<0.01$, +++ $p<0.001$).

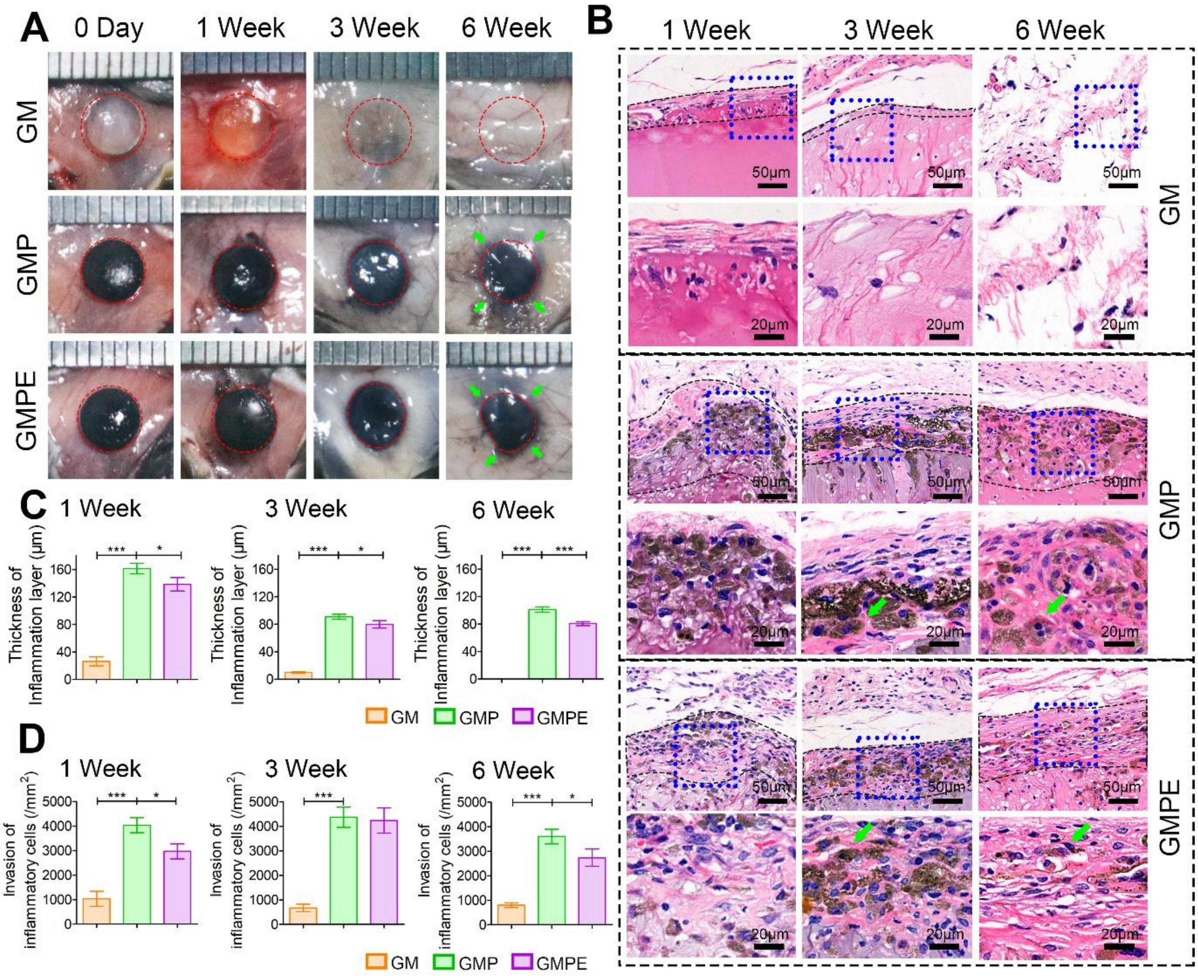

Figure S6 In vivo biodegradability of implanted hydrogels. (**A**) The degradation rate of GMPE and GMP hydrogels was significantly slower than that of the GM hydrogel after subcutaneous implantation. (**B**) HE staining shows the degradation process and inflammation of each hydrogel after implantation. Green arrows indicate where cells have phagocytosed PPy nanoparticles. Scale bars, 50 µm. (**C**) The thickness of the fibrotic capsule was qualified at 1, 3 and 6 weeks post-implantation (n=3). (**D**) Quantification of invasive inflammatory cells at 1-, 3-, and 6-week time points (n=3). Statistical differences were determined using an ANOVA with Bonferroni's multiple comparison test (* p<0.05, ** p<0.01, ***p<0.001).

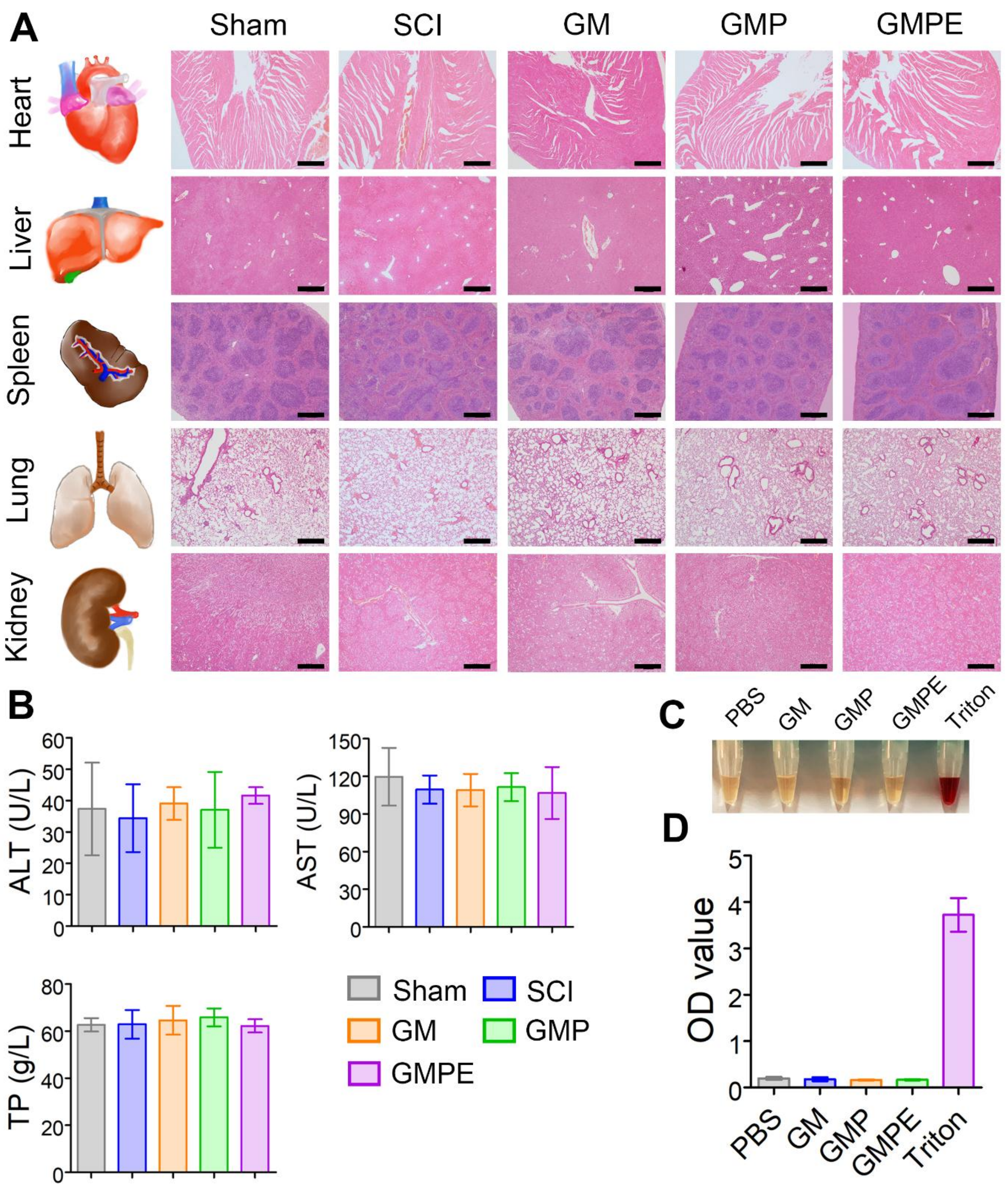

Figure S7 In vivo biocompatibility of implanted hydrogels. (**A**) HE staining indicating normal morphology in the heart, liver, spleen, lung, and kidney tissues from each treatment group. Scale bars, 500 μm. (**B**) The level of ALT, AST, TP in serum were similar in all treatment groups, indicating hydrogels did not cause systemic toxicity (n=3). (**C**) Photograph of serum extracted from whole blood co-cultured with each hydrogel. Samples were light yellow in color and similar to that of the PBS control group, while the Triton-100X group was bright red, indicating its hematolysis. (**D**) The serum OD values in GM, GMP and GMPE groups were similar to the value in the PBS group, while all were significantly lower than that of the Triton-100X group (n=3). Statistical differences were determined using an ANOVA with Bonferroni's multiple comparison test (* $p<0.05$, ** $p<0.01$, *** $p<0.001$).

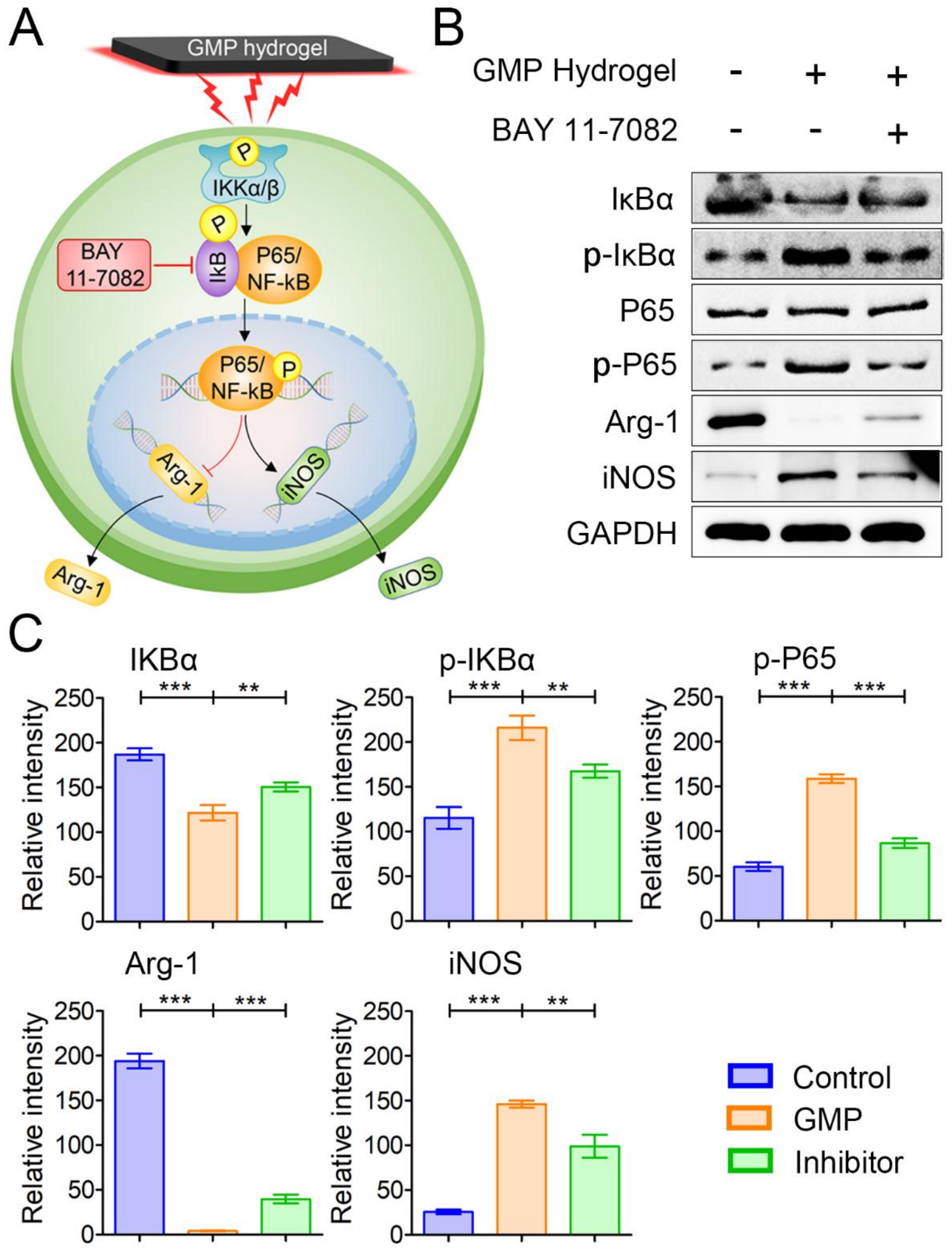

Figure S8 Microglia M1/M2 polarization was evaluated after p-IκBα was inhibited with BAY 11-7082 treatment. (**A**) Illustration of the target site for BAY 11-7082 inhibition of p-IκBα activity. (**B**) After p-IκBα was selectively inhibited, IκBα, p-IκBα, P65, p-P65, iNOS, and Arg-1 protein expression was evaluated by WB. (**C**) The graphs illustrate the quantification of protein bands intensity (n=3). Statistical differences were determined using an ANOVA with Bonferroni's multiple comparison test (* $p<0.05$, ** $p<0.01$, ***$p<0.001$).

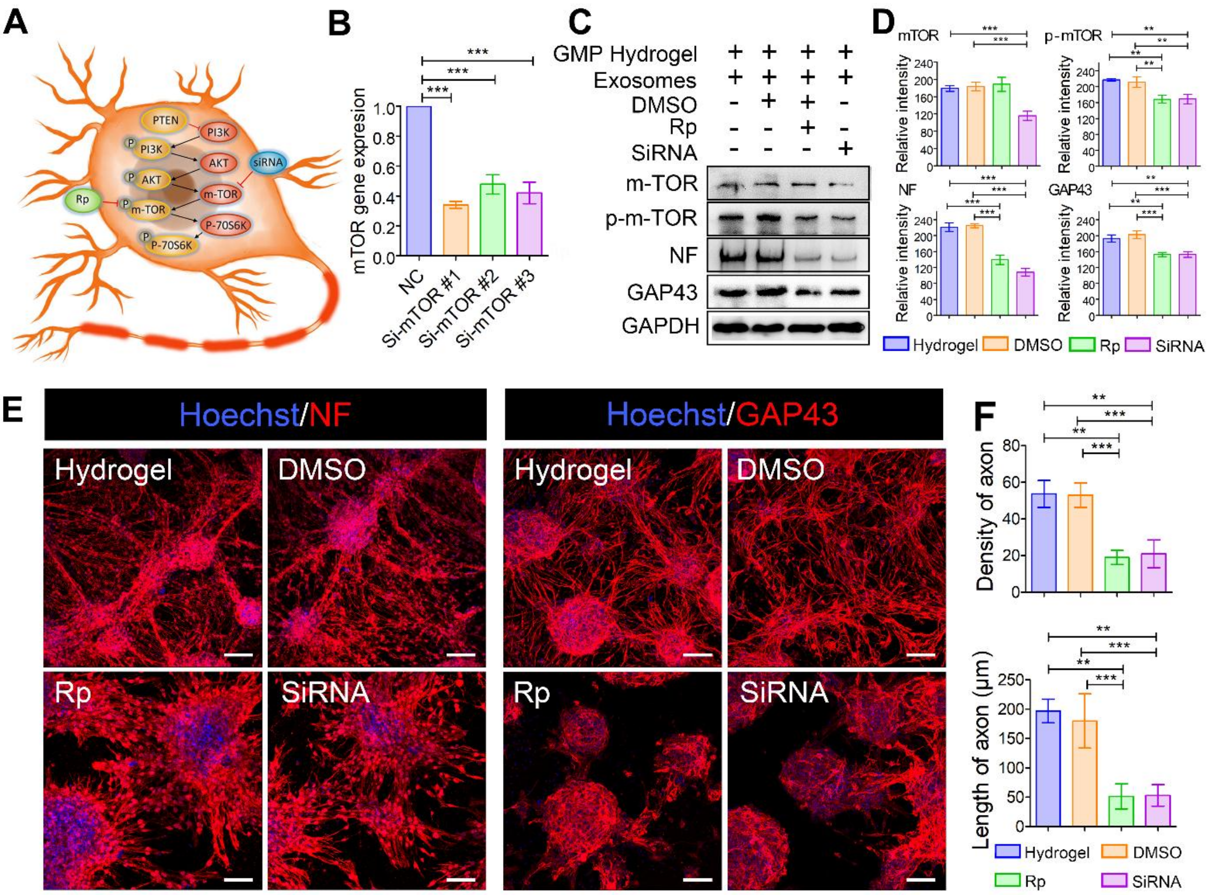

Figure S9 NSC axon outgrowth on GMPE hydrogels after selective p-mTOR inhibition. (**A**) Illustration of the effect of Rp and siRNA on mTOR protein. (**B**) RT-qPCR of m-TOR expression after knockdown by three siRNAs (n=3). (**C**) mTOR, p-mTOR, NF and GAP43 protein expression was evaluated 7 days after mTOR was selectively inhibited. (**D**) Graph of protein expression in the experimental growth conditions (n=3). (**E**) Axon-related protein localization in NSCs after mTOR inhibition. Red IF represents the NF or GAP43, respectively. Scale bars, 100 μm. (**F**) Axon density (n=5) and length were quantified (n=11). Statistical differences were determined using an ANOVA with Bonferroni's multiple comparison test (* $p<0.05$, ** $p<0.01$, ***$p<0.001$).

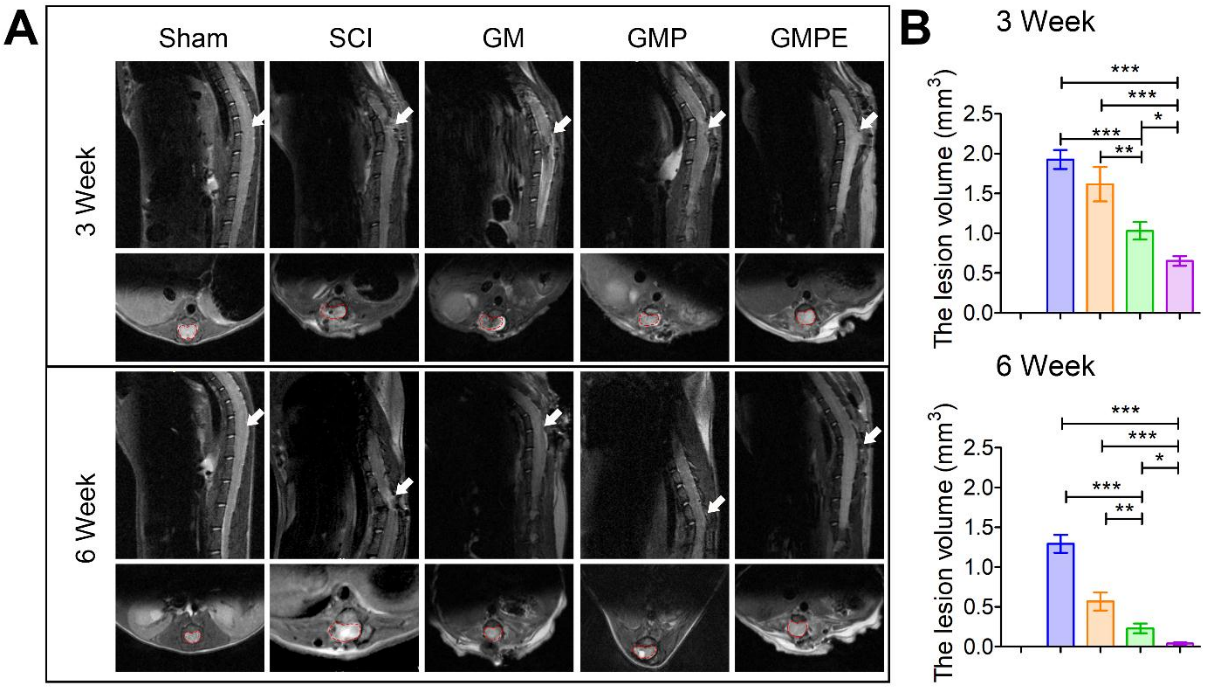

Figure S10 Spinal cord pathology after SCI at 3 and 6 weeks. (**A**) Conventional MRI was used to assess changed in spinal cord pathology in sham and hydrogel implantation after SCI. Write arrows indicate the SCI site. The sites of spinal cord in the transverse section were marked in red dotted frame. (**B**) Quantitative analysis of lesion volume (n=3) at 3- and 6-weeks. Statistical differences were determined using an ANOVA with Bonferroni's multiple comparison test (* $p<0.05$, ** $p<0.01$, ***$p<0.001$).

Table S1. Serum and cellular contaminants were negative in the BMSC-derived exosomes detected by the proteomic mass spectrometry.

Source	**Protein**
Nucleus	histones
Mitochondria	Cytochrome C
Golgi	GM130
ER	calnexin
Culture media	albumin

Table S2. Primer sequences of each gene was showed below.

Target	Forward	Reverse
GAPDH	AGGTCGGTGTGAACGGATTTG	GGGGTCGTTGATGGCAACA
Tuj-1	TCACGCAGCAGATGTTCGAT	GTGGCGCGGGTCACA
GFAP	CCTGAGAGAGATTCGCACTCAA	CTCCTCTGTCTCTTGCATGTTACTG
MBP	ACAGAGACACGGGCATCCTT	CACCCCTGTCACCGCTAAAG
NF	GTTCCGAGTGAGGTTGGACC	CCGCCGGTACTCAGTTATCTC
GAP43	ATAACTCCCCGTCCTCCAAGG	GTTTGGCTTCGTCTACAGCGT
IL10	CTTACTGACTGGCATGAGGATCA	GCAGCTCTAGGAGCATGTGG
IL6	CTTCCATCCAGTTGCCTTCT	CTCCGACTTGTGAAGTGGTATAG
iNOS	GTTCTCAGCCCAACAATACAAGA	GTGGACGGGTCGATGTCAC
ARG	CTCCAAGCCAAAGTCCTTAGAG	GGAGCTGTCATTAGGGACATCA
TNFα	CGAGTGACAAGCCTGTAGCC	ACAAGGTACAACCCATCGGC
mTOR	CCACTCTCTGACCCAGTTCG	GATGCCAAGACACAGTAGCG
MiR-29a	cagtagcaccatctgaaatcg	tccagtttttttttttttttaaccga
MiR-21	gcagtagcttatcagactgatg	ggtccagtttttttttttttttcaac
MiR-199a	cagacagtagtctgcacattg	gtccagtttttttttttttttaaccaa
MiR-29c	cagtagcaccatttgaaatcg	tccagtttttttttttttttaaccga
MiR-26a	gcagttcaagtaatccaggatag	gtccagtttttttttttttttagcct
MiR-20a	gcagtaaagtgcttatagtgcag	gtccagtttttttttttttttctacct
MiR-9-5p	gcagtctttggttatctagctgt	ggtccagtttttttttttttttcatac
MiR-182	gtttggcaatggtagaactca	ccagtttttttttttttttcggtgt
MiR-128	gtcacagtgaaccggtct	ggtccagtttttttttttttttaaagaga
MiR-133b	cagtttggtccccttcaac	gtccagtttttttttttttttagctg
MiR-431	tcttgcaggccgtca	ccagtttttttttttttttgcatga
MiR-124	aggcacgcggtga	ccagtttttttttttttttggcattc
MiR-145a	ggtccagttttcccagga	ccagtttttttttttttttagggattc
MiR-146a	gcagtgagaactgaattcca	ggtccagtttttttttttttttaacc
MiR-148b	agtcagtgcatcacagaac	ggtccagtttttttttttttttacaaag
MiR-138	agagctggtgttgtgaatc	gtttttttttttttttcggcctga
MiR-338	gcagtccagcatcagtga	gtccagtttttttttttttttcaaca
MiR-219a	cagtgattgtccaaacgca	ggtccagtttttttttttttttagaattg
MiR-1a	cgcagtggaatgtaaagaagt	ggtccagtttttttttttttttatacatac
MiR-99a	cagaacccgtagatccga	tccagtttttttttttttttcacaaga
MiR-181a	cattcaacgctgtcggt	gtccagtttttttttttttttactcac
MiR-411	cagtagtagaccgtatagcgt	ggtccagtttttttttttttttcgt

Table S3. Sequences of three siRNAs were showed below.

Target	Sense	Antisense
Si-mTOR1	5'-GAACUCGCUGAUCCAGAUG dTdT-3'	5'-CAUCUGGAUCAGCGAGUUC dTdT-3'
Si-mTOR2	5'- GGUCAUGCCCACAUUCCUU dTdT-3'	5'-AAGGAAUGUGGGCAUGACC dTdT-3'
Si-mTOR3	5'-GCAUAUGGCCGAGAUUUAA dTdT-3'	5'-UUAAAUCUCGGCCAUAUGC dTdT-3'

Table S4. The comparison of conductivity

Conductive hydrogel	Conductivity(S/cm)	reference
GelMA/rGO	8.7×10^{-3}	[37]
GelMA-BP@PDA0.3	1.9×10^{-3}	[38]
PPy/GelMA2	1×10^{-2}	[39]
GelMA/PCL@Gel-PPy	3.1×10^{-3}	[40]
DA-PPy-GP	7×10^{-4}	[41]
